# Multifaceted Regulation of Translational Readthrough by RNA Replication Elements in a Tombusvirus

**DOI:** 10.1371/journal.ppat.1002423

**Published:** 2011-12-08

**Authors:** Peter A. Cimino, Beth L. Nicholson, Baodong Wu, Wei Xu, K. Andrew White

**Affiliations:** Department of Biology, York University, Toronto, Ontario, Canada; University of California, Riverside, United States of America

## Abstract

Translational readthrough of stop codons by ribosomes is a recoding event used by a variety of viruses, including plus-strand RNA tombusviruses. Translation of the viral RNA-dependent RNA polymerase (RdRp) in tombusviruses is mediated using this strategy and we have investigated this process using a variety of in vitro and in vivo approaches. Our results indicate that readthrough generating the RdRp requires a novel long-range RNA-RNA interaction, spanning a distance of ∼3.5 kb, which occurs between a large RNA stem-loop located 3'-proximal to the stop codon and an RNA replication structure termed RIV at the 3'-end of the viral genome. Interestingly, this long-distance RNA-RNA interaction is modulated by mutually-exclusive RNA structures in RIV that represent a type of RNA switch. Moreover, a different long-range RNA-RNA interaction that was previously shown to be necessary for viral RNA replicase assembly was also required for efficient readthrough production of the RdRp. Accordingly, multiple replication-associated RNA elements are involved in modulating the readthrough event in tombusviruses and we propose an integrated mechanistic model to describe how this regulatory network could be advantageous by (i) providing a quality control system for culling truncated viral genomes at an early stage in the replication process, (ii) mediating cis-preferential replication of viral genomes, and (iii) coordinating translational readthrough of the RdRp with viral genome replication. Based on comparative sequence analysis and experimental data, basic elements of this regulatory model extend to other members of Tombusviridae, as well as to viruses outside of this family.

## Author Summary

Viruses use many different strategies to produce their proteins and some viral proteins are made with terminal extensions that confer unique properties. The polymerase that replicates the RNA genomes of tombusviruses is an extended version of another viral protein and is generated by a process called translational readthrough. We have determined the regulatory mechanism that modulates the production of this viral polymerase. Our results show that control of the readthrough process is complex and involves both local structures and long-range interactions within the viral genome. This system is also integrated with viral RNA replication elements and this allows the virus to coordinate polymerase production with genome replication. This regulatory scheme appears to represent a common tactic used by a variety of viruses.

## Introduction

RNA viruses utilize a vast array of coding and gene expression strategies to maximize the utility and regulation of their genomes [1]–[4]. One such tactic used by different viruses is the recoding of stop codons as sense codons, which allows ribosomes to continue translating past the normal termination point of an open reading frame (ORF) [2], [5]. This so-called stop codon readthrough (RT) event occurs inefficiently, thus the C-terminally extended, or RT, product is made at much lower levels than the pre-RT product [2]. Bypass of termination codons is made possible by aminoacylated suppressor tRNAs, which have anticodons that can base pair with stop codons and allow ribosomes to introduce their cognate residues into the nascent polypeptide chain [6]. Subsequent to the recoding event, normal translation of the ORF resumes with reading of the ensuing inframe sense codons.

RNA sequences and/or structures next to recoded stop codons have been shown to be important for RT [7], [8]. For some plant RNA viruses, the linear sequence 3'-adjacent to the stop codon is important for efficient RT, *e.g.* Tobacco mosaic virus (TMV) [9]. Conversely, 3'-adjacent RNA secondary structures necessary for RT have, thus far, not been shown to be functionally relevant for any plant virus. However, recent comparative sequence analyses based on the identification of coding regions that have lower than expected levels of synonymous codon substitutions have revealed potential RNA secondary structures 3'-proximal to the RT sites in a variety of plus-strand RNA plant viruses, including genera in the family Tombusviridae [8].

Tombusvirus is the type genus of the family Tombusviridae, which currently includes eight genera [10], [11]. Of these genera, seven express their RNA-dependent RNA polymerase (RdRp) by RT, whereas one, Dianthovirus, uses translational frameshifting [12]. Dianthoviruses use the most common form of frameshifting that involves the shifting of a translating ribosome from the 0 reading frame to the -1 reading frame, where it resumes translation of the nascent polypeptide [13], [14]. Like RT, frameshifting also involves sequences and/or structures 3'-proximal to the frameshift site [14]–[16].

**Figure 1:**
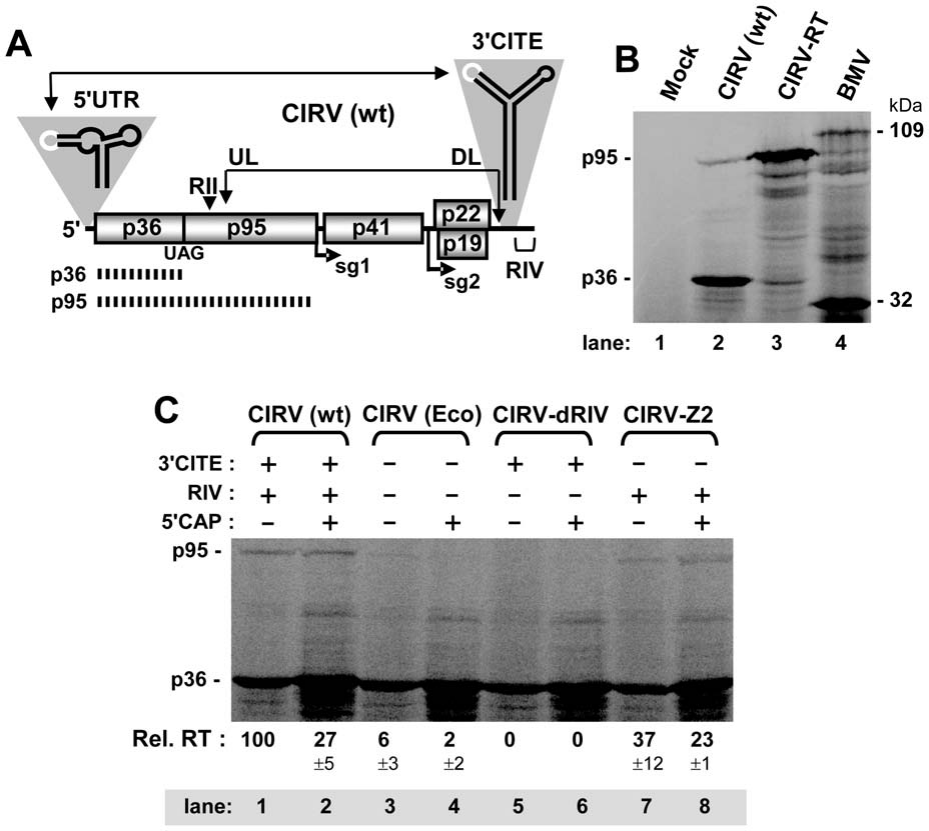
In vitro translation of the CIRV genome assessing the role of the 3'UTR in RT. (**A**) Schematic linear representation of the CIRV RNA genome with boxes representing encoded proteins and a thick horizontal line representing non-coding regions. p36 and its RT product, p95 (depicted by the hashed lines), are translated directly from the viral genome. Initiation sites for sg mRNA1 (sg1) and sg2 are indicated below the genome. Relevant RNA structures in the 5' and 3'UTRs, the T-shaped domain and 3'CITE, respectively, are shown schematically above the genome with complementary adapter sequences shown in white. Double-headed arrows connect RNA sequences that base pair over long distances, as shown for the 5'UTR-3'CITE interaction. An additional long-range RNA-RNA interaction, the UL-DL interaction, unites two important RNA replication elements, RII and RIV, so that replicase complex assembly can occur. (**B**) SDS-10%PAGE analysis of proteins translated from the CIRV genome. The mock lane consists of a translation reaction in the absence of RNA, while the BMV lane contains Brome mosaic virus RNAs 1 and 3, that template translation of proteins of 109 and 32 kDa, respectively, which served as molecular mass markers. Wt and mutant CIRV genomes are indicated above the center lanes and the positions of the CIRV p36 and p95 are indicated to the left. Protein products in this and all subsequent in vitro translation experiments were generated by translating 0.5 pmol of viral genome in wheat germ extract (wge) for 1 hr at 25°C, and, unless specified, the messages were uncapped. (**C**) SDS-10%PAGE analysis of RT for CIRV genomes containing various modifications to the 3'UTR. Note, the KOAc concentration in this and subsequent in vitro translation assays was optimized for efficient readthrough, not for 3'CITE-dependent translation. The presence or absence of the 3'CITE, RIV or a cap structure in the genome is indicated above each lane as a + or -, respectively. In this and subsequent experiments, the p95:p36 ratio was determined for each lane, and the relative RT percentages (Rel. RT) below each lane correspond to means (± standard error) from three independent experiments that were normalized to the p95:p36 ratio for the wt genome, set at 100.

Within Tombusviridae, members of the genus Tombusvirus, including the species Carnation Italian ringspot virus (CIRV) [17], are among the best characterized molecularly [11]. These viruses possess plus-strand RNA genomes of ∼4.8 kb in length and encode five functional proteins [11]. In CIRV, the 5'-proximally-encoded pre-RT product p36 and its RT-polypeptide p95, the RdRp, are translated directly from the viral genome [18], and both of these viral proteins are essential for viral RNA replication [11] (Figure 1A). In vivo, the tombusvirus pre-RT product accumulates at ∼20-fold greater amounts than the RdRp [19], consistent with RT being inefficient in plant infections. As tombusvirus RNA genomes are neither 5'-capped nor 3'-polyadenylated, they rely on a 3' cap-independent translational enhancer (3'CITE) located in their 3' untranslated region (UTR), which assists with recruitment of the translational machinery [18], [20]. Initiation of translation at the 5'-end of the viral genome is mediated by the 3'CITE interacting with the genomic 5'UTR via a long-range base pairing interaction [18] (Figure 1A). This interaction is proposed to direct delivery of 3'CITE-recruited translational machinery to the 5'-proximal site of translation initiation [20]. Additional long-range RNA-RNA interactions have been identified in tombusvirus genomes that mediate other important viral processes such as, transcription of viral subgenomic (sg) mRNAs that template translation of 3'-proximal viral proteins [21]-[23] or assembly of the RNA replicase complex that replicates the viral genome [24]. In the latter case, two essential RNA replication elements, region II (RII) in the p95 RT region [25], [26] and RIV in the 3'UTR [27]–[29], are united by an upstream linker-downstream linker (UL-DL) RNA-RNA base pairing interaction to create an RII-RIV RNA platform for replicase complex assembly [24] (Figure 1A). The 5'UTR-3'CITE, the UL-DL, and three other transcription-related long-range RNA-RNA interactions comprise a vast RNA-based regulatory network that underscores the complexity and high level of organization of the global structure of these viral genomes [4], [24].

In the present study, we used CIRV as a model tombusvirus to study the regulation of RT. Our results revealed that this process is modulated by a novel RNA-based system that includes two long-range RNA-RNA interactions and an RNA switch. The data show a direct link between RT and viral RNA replication, which we propose is important for regulating and coordinating these two processes. Importantly, components of this mechanism are conserved in other members of Tombusviridae and related viruses.

## Results

### 3'-proximal sequences are required for translational RT generating p95

An in vitro wheat germ extract (wge) translation system and subsequent sodium dodecyl sulfate-polyacrylamide gel electrophoresis (SDS-PAGE) were utilized to assess translational RT leading to production of p95 from the CIRV RNA genome. Translation of the wt CIRV genome resulted in the abundant accumulation of p36 and a small amount of a larger product with an estimated molecular mass consistent with that of p95 (Figure 1B, lane 2). To confirm the identity of the larger product as p95, we analyzed a CIRV genomic mutant, termed CIRV-RT, which had its p36 stop codon substituted with a tyrosine sense codon. The major product from CIRV-RT co-migrated with the proposed p95 readthrough product and there was a notable reduction in the accumulation of p36 (Figure 1B, compare lane 2 with 3). These results are consistent with the assigned identities of the major and minor bands generated from the wt CIRV genome as p36 and its RT product p95, respectively. The RT efficiency in this in vitro system was calculated to be 0.81% ± 0.05.

We next investigated whether the 3'UTR contributed to RT levels in vitro. Relative RT efficiency for each genome was calculated as a ratio of p95/p36 accumulation, with that for wt CIRV set at 100%. This approach also provided an integrated correction for potential differences in general message decay and/or translation initiation rates, because each RT value was calculated relative to its cognate p36 level. When both the 3'CITE and RIV were deleted in mutant CIRV(Eco), the RT efficiency fell to ∼6% (Figure 1C, lane 3). 5'-capping of CIRV(Eco) improved overall translation efficiency, as determined by the increase in p36, however RT was still severely compromised (Figure 1C, lane 4). Interestingly, deleting the 3'-terminal RIV in mutant CIRV-dRIV eliminated detectable RT, regardless of the presence or absence of a 5'-cap (Figure 1C, lane 5 and 6). Finally, deletion of the 3'CITE, while maintaining RIV, in mutant CIRV-Z2 resulted in retained RT, albeit at lower than wt levels (Figure 1C, lanes 7 and 8). Thus, moderate to wt levels of RT were observed when RIV was present, implicating this RNA replication element in activation of RT.

### A long-range RNA-RNA interaction is required for RT

**Figure 2:**
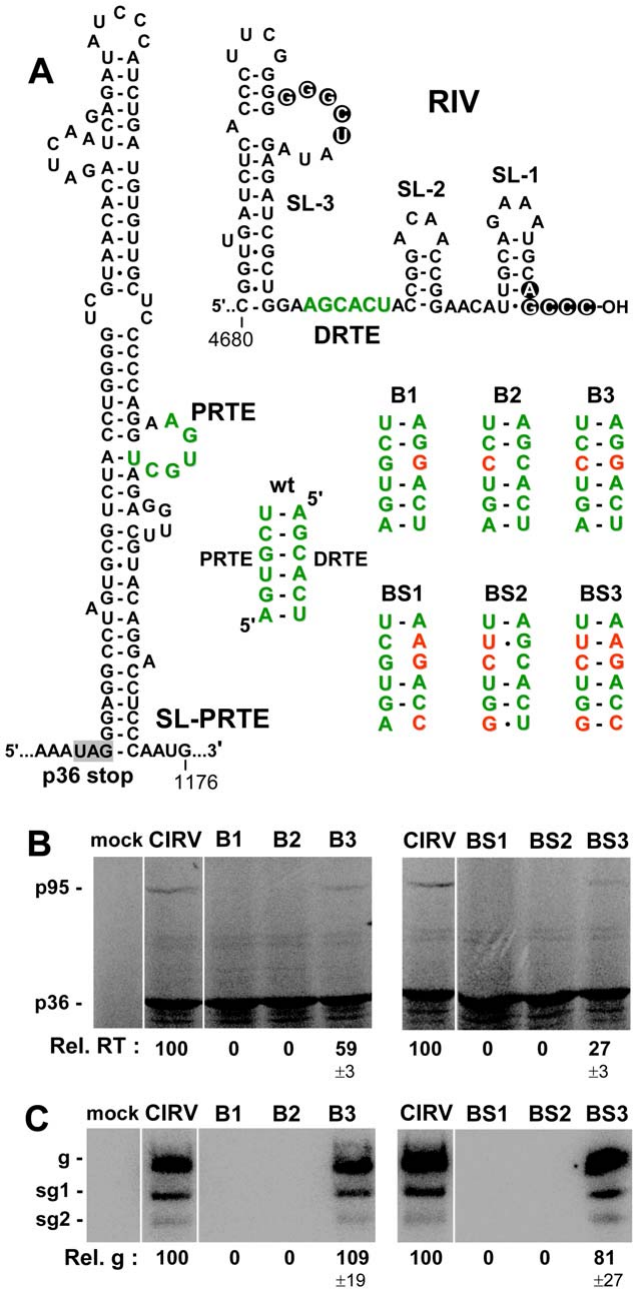
Role of the PRTE-DRTE interaction in mediating RT and genome replication. (**A**) Predicted RNA secondary structures of SL-PRTE and RIV, with the complementary PRTE and DRTE sequences shown in green. Nucleotides involved in a functional intra-RIV interaction between an internal loop in the replication silencer element SL-3 and the 3'-terminal sequence are shown in white within circles. Wt and mutant PRTE-DRTE interactions are shown with substituted nucleotides depicted in red. (**B**) In vitro translation in wge of CIRV genomes containing various mutations disrupting and restoring the PRTE-DRTE interaction as shown in panel A. (**C**) Northern blot analysis and quantification of genomic plus-strand accumulation 22 hr post-transfection of plant protoplasts. The viral genomes analyzed are indicated above each lane and correspond to those depicted in panel A. The positions of the genomic (g) and subgenomic RNAs (sg1 and sg2) are indicated to the left of the blot. The relative values for viral genome accumulation (Rel. g), in this and subsequent experiments, correspond to means (± standard error) from three independent experiments and were normalized to the accumulation of wt genomic RNA levels, set at 100.

Sequences and structures 3'-adjacent to RT sites are often instrumental in facilitating the RT process [6], [7]. Analysis of this region in the CIRV genome by mfold [30], [31] and selective 2'-hydroxyl acylation analyzed by primer extension (SHAPE) [32] suggested the presence of an extended RNA stem-loop (SL) structure, termed SL-proximal readthrough element (SL-PRTE) (Figure 2A and Figure S1). Mfold analysis computationally predicts the lowest free-energy secondary structure for a given RNA sequence [30], [31], while SHAPE analysis allows for the identification of nucleotides in an RNA structure that are flexible in solution and thus likely single stranded [32]. The predicted SL-PRTE structure was also consistent with the results of comparative RNA secondary structure analysis of sequenced species in the genus Tombusvirus (Figure S2). Consequently, it represented a good candidate structure for contributing to RT. However, as RIV was shown to be required for efficient RT (Figure 1C), we considered the possibility that RIV may somehow communicate with SL-PRTE. Indeed, close analysis of RIV secondary structure revealed a 6 nt long sequence in a predicted single-stranded RNA region, termed the distal readthrough element (DRTE), which was complementary to sequence in one of the bulges in SL-PRTE, termed the proximal readthrough element (PRTE) (Figure 2A).

Two sets of CIRV genomic compensatory mutants designed to disrupt and then restore the putative PRTE-DRTE interaction were generated. The first set targeted a single pair in the proposed interaction (mutant genomes B1, B2 and B3), while the second set targeted three pairs simultaneously (BS1, BS2 and BS3) (Figure 2A). Importantly, the nucleotide substitutions in the PRTE did not change the corresponding codons specifying p95. In wge, mutations that were predicted to weaken the interaction abolished detectable RT, whereas restoration of the interaction with alternate base pairs partially restored RT (Figure 2B). A similar functional correlation was also observed when the same mutant genomes were transfected into plant protoplasts and viral genome accumulation was monitored by Northern blotting (Figure 2C). These results indicate that a long-range base pairing interaction between the PRTE and DRTE is required for both efficient RT in vitro and robust genome accumulation in vivo.

### Physical evidence for the PRTE-DRTE interaction

**Figure 3:**
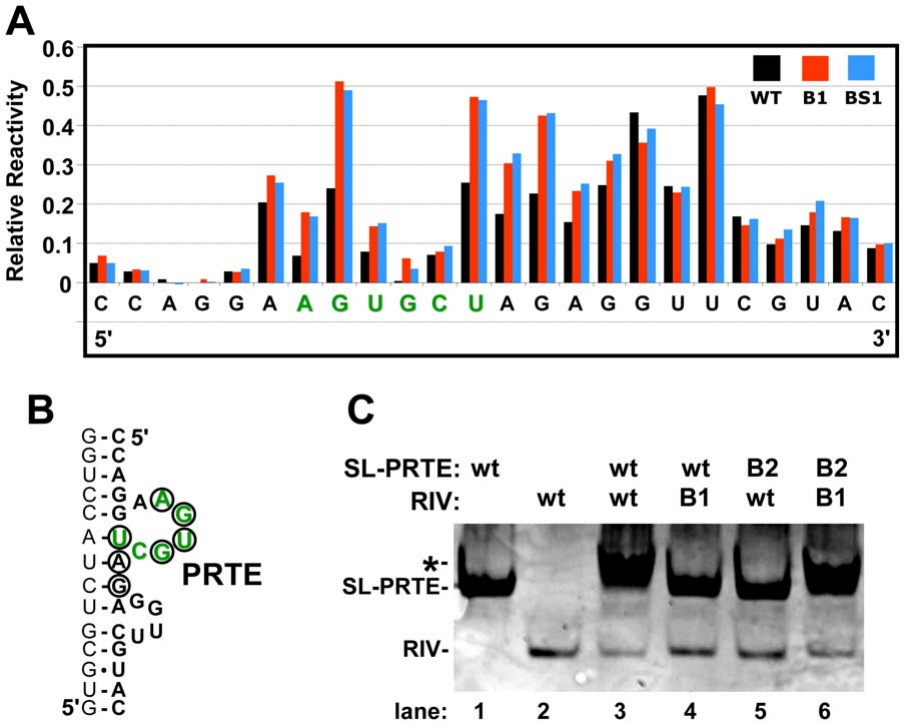
Structural analysis of the PRTE-DRTE interaction. (**A**) SHAPE analysis of the PRTE and its flanking sequence. Sequence corresponding to the PRTE is shown in green. Relative reactivity of each nucleotide is plotted graphically, with larger values corresponding to increased flexibility. The genomic mutants assayed are indicated in the key at the top right and correspond to those shown in Figure 2A. (**B**) Predicted secondary structure of the PRTE and its flanking regions showing residues in the mutants with notably increased reactivity (circled), as determined from the results in panel A. (**C**) RNA-RNA EMSA assessing SL-PRTE binding to RIV using wt and B-series compensatory mutants shown in Figure 2A. Equimolar amounts of unlabeled RNA fragments were incubated at 25°C for 30 min and the mixtures were then separated in a nondenaturing 15% acrylamide gel and stained with ethidium bromide to allow for visualization of the RNAs (the negative image is shown). The left-most lanes show the positions of free SL-PRTE and RIV, and the asterisk depicts the position of the upward shift observed when binding occurs.

To gain additional support for the proposed RNA-based interaction, physical studies were performed. The first was carried out in the context of the wt and mutant CIRV genomes and examined the flexibility of the PRTE region using SHAPE (Figure 3A). If the substitutions made in the DRTE in mutants B1 and BS1 were disrupting the PRTE-DRTE interaction one would predict increased flexibility in the complementary PRTE region. SHAPE analysis of this region in B1 and BS1 indicated higher relative levels of flexibility compared to wt CIRV for several of the residues in the PRTE, as well as some of the adjacent nucleotides (Figure 3A and B), which is consistent with disruption of the interaction. In contrast, more distal flanking residues that were not predicted to participate in the long-range interaction served as useful internal controls and showed comparable levels of SHAPE reactivity. Similar SHAPE analysis at the DRTE was not possible as it is located too close to the 3'-end to allow for oligonucleotide priming.

To complement the results above, RNA-RNA electrophoretic mobility shift assays were performed using wt and mutant forms of SL-PRTE and RIV. When wt versions of the two RNA fragments were combined, there was a notable upward shift in mobility and smearing of the SL-PRTE band when compared to SL-PRTE alone (Figure 3C, compare lane 3 with 1) along with a reduction in RIV (Figure 3C, compare lane 3 with lane 2). Conversely, the wt and mutant combinations showed no evidence of an interaction (Figure 3C, lanes 4 and 5). The mixture of the two mutant fragments, where complementarity was restored, yielded a shift and smear similar to the wt-wt combination (Figure 3C, compare lane 6 with 3). When combined with the SHAPE data, these results support the concept that the PRTE interacts directly with the DRTE via base pairing.

### In vivo evidence for an RT defect in viral genomes containing a disrupted PRTE-DRTE interaction

**Figure 4:**
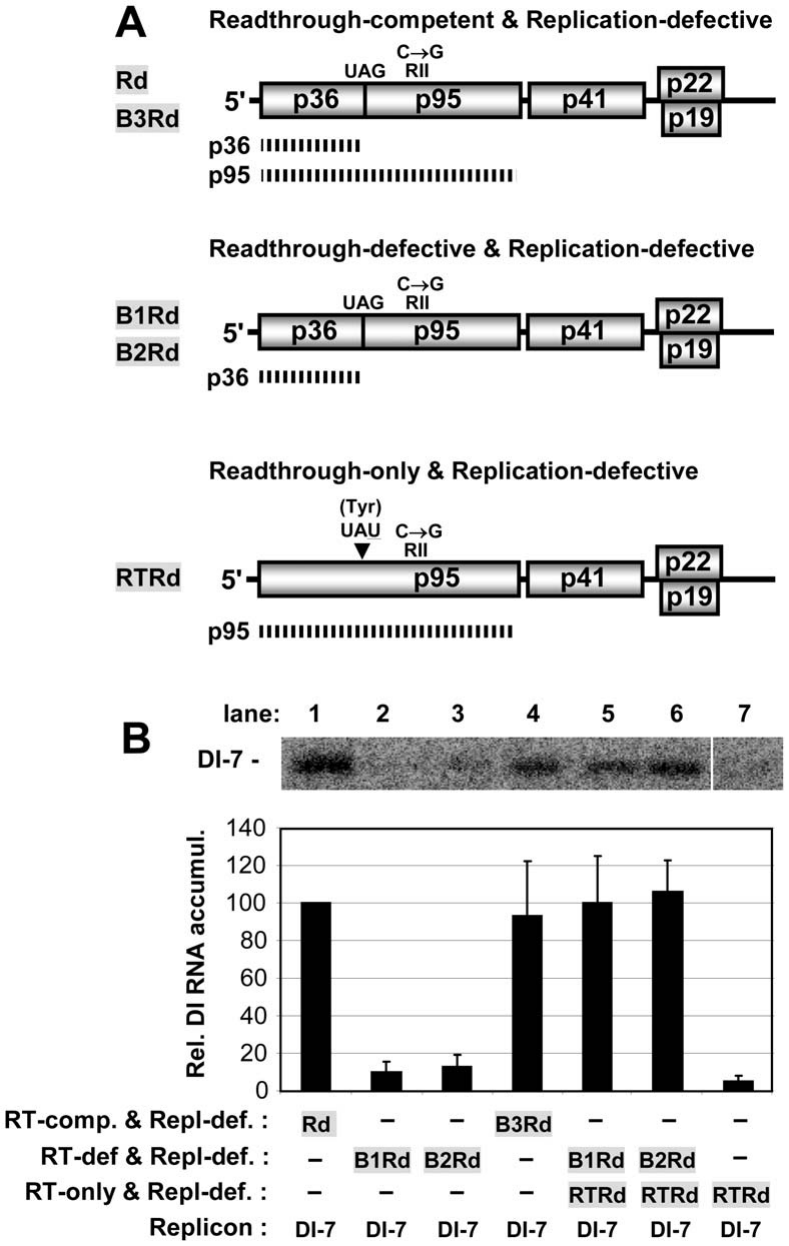
Analysis of RT using replication-defective genomes and a DI RNA replicon reporter. (**A**) Schematic diagrams of non-replicating CIRV genomes used to assess p95 production. The functional properties of the different mutants are shown above the genomes and the names of the mutants are shown to the left. The C-to-G mutation in RII that renders all the genomes replication defective is shown above each genome. At the top, the mutants Rd and B3Rd are able to produce both p36 and p95, as indicated by the two hashed lines corresponding to these proteins. In the middle, mutants B1Rd and B2Rd are RT-defective and can produce only p36. Additional modifications in the PRTE and DRTE in mutants B1Rd, B2Rd, and B3Rd correspond to those in mutants B1, B2, and B3, respectively, as shown in Figure 2A. At the bottom, RTRd contains a G-to-U mutation in its stop codon, which changes it to a Tyr codon so that only p95 is produced. (**B**) Analysis and quantification of DI-7 accumulation when cotransfected with non-replicating CIRV genomes in plant protoplasts. The CIRV genomes cotransfected with DI-7 are indicated below each bar in the graph. DI-7 RNAs were analyzed by Northern blotting (top) 22 hr post-transfection. The relative values for DI-7 accumulation shown in the bar graph, in this and other experiments with DI RNA replicons, correspond to means (± standard error) from three independent experiments and were normalized to the accumulation of DI-7 cotransfected with Rd, set at 100.

We next sought to acquire experimental evidence that the PRTE-DRTE interaction was able to mediate RT in vivo. However, we were not able to demonstrate PRTE-DRTE-dependent RT in vivo using a dual luciferase reporter mRNA construct [33] with the viral 5'UTR, SL-PRTE region and 3'UTR positioned, respectively, at the 5'-end, the intervening rluc/fluc region and the 3'-end of the message (data not shown), suggesting that the viral genomic context was critical for function. Consequently, an alternative in vivo approach was designed that maintained the viral genomic context. We first rendered the wt CIRV genome and B-series genomic mutants replication-defective by introducing a C-to-G substitution in an essential RNA replication element in RII (Figure 4A), a strategy used previously [26]. Though unable to replicate itself, the replication-defective wt genome, Rd, was still able to provide sufficient p36 and p95 for replication of a cotransfected small non-coding CIRV-derived RNA replicon [34], termed DI-7 (Figure 4B, lane 1). In contrast, replication-defective versions of mutants B1 and B2, termed B1Rd and B2Rd, did not support efficient replication of DI-7 in trans (Figure 4B, lanes 2 and 3). Conversely, replication-defective B3Rd, with the PRTE-DRTE interaction restored, was able to effectively complement DI-7 accumulation (Figure 4B, lane 4). If the inability of B1Rd and B2Rd to amplify DI-7 was based on an RT defect that caused insufficient levels of p95 (Figure 4A), then it should be possible to rescue DI-7 replication by providing p95 in trans from an alternate source. To this end, a replication-defective version of CIRV-RT (Figure 1B), RTRd, was created that would efficiently produce p95 (Figure 4A). Cotransfection of RTRd alone with the replicon did not direct efficient amplification of DI-7, because both p36 and p95 are required for viral RNA replication (Figure 4B, lane 7). However, when either B1Rd or B2Rd was cotransfected with RTRd and DI-7, there were relatively high levels of DI-7 accumulation (Figure 4B, lanes 5 and 6). This rescue was dependent on the RdRp activity of p95 provided by RTRd, as mutation of the essential GDD motif of p95 in RTRd prevented complementation (Figure S3). These data are consistent with the in vitro data indicating that RT, but not translation of p36, is defective in B1 and B2 (Figure 2B), because the inability of B1Rd or B2Rd to efficiently amplify the DI-7 replicon could be rescued by providing functional p95 in trans from RTRd (Figure 4B).

### The long-range interaction is important, but not essential, for genome replication

**Figure 5:**
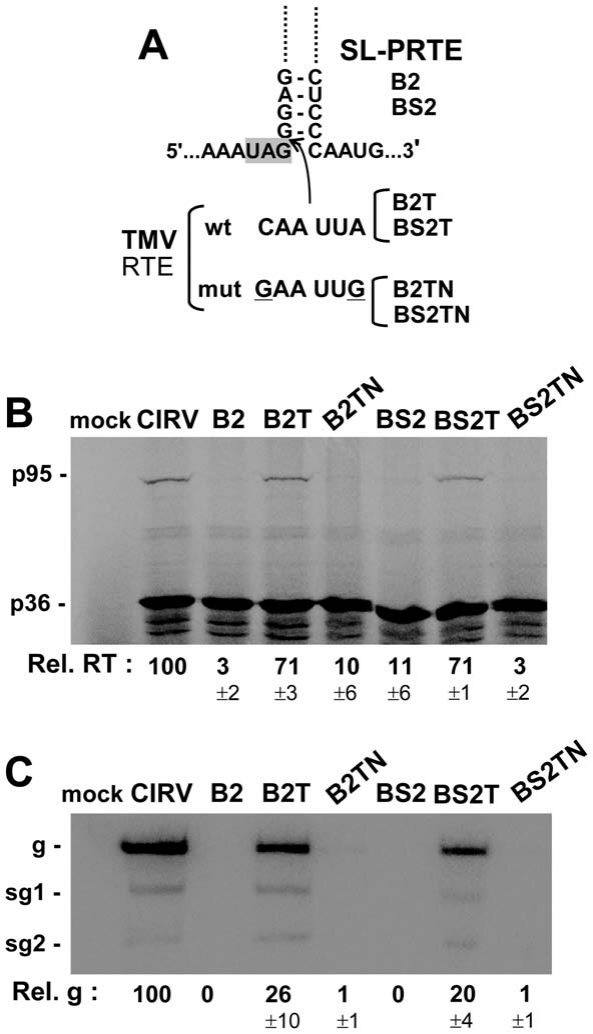
Restoring RT and genome replication with a heterologous RT sequence. (**A**) At the top, a portion of the SL-PRTE structure is shown with the insertion site (arrow) of wt and mutant forms of the 6 nt long TMV RTE sequence. Wt and mutant TMV RTE sequences in corresponding genomic mutants are shown below. Substitutions in the mutant TMV RTE are underlined. (**B**) In vitro translation in wge of CIRV genomes containing mutations disrupting the PRTE-DRTE interaction (B2 and BS2) as well as the wt TMV RTE (B2T and BS2T) or the mutated TMV RTE (B2TN and BS2TN). (**C**) Northern blot analysis of wt and mutant CIRV genomes in protoplasts and quantification of plus-strand viral genome accumulation.

Having established a requirement for the PRTE-DRTE interaction for RT, we wondered if the long-range interaction itself, independent of its RT function, was important for genome replication. In order to investigate this possibility, RT needed to be uncoupled from the long-range interaction. To do this, the linear TMV readthrough element (RTE), CAAUUA, was inserted just downstream of the p36 stop codon in RT-defective B2 and BS2 (that contained disrupted PRTE-DRTE interactions), thereby creating B2T and BS2T, respectively (Figure 5A). Introduction of the wt TMV RTE in the RT-defective mutants led to relatively efficient recovery of RT in vitro, while adding a mutated TMV RTE did not (Figure 5A, B). Moreover, there was readily detectable replication of the wt TMV RTE-containing genomes when they were transfected into protoplasts (Figure 5C). However, the level of viral genome accumulation was not restored to that of wt in either case, suggesting that although the long-range interaction is not essential for genome replication, it may contribute additional non-RT functions important for efficient virus genome reproduction. Conversely, it is possible that the lower than wt levels of RT observed for B2T and BS2T (Figure 5B) could have contributed to the observed reduced genome accumulation in protoplasts (Figure 5C). This prospect, however, seems less likely as even lower levels of RT were observed for the mutants B3 and BS3, yet near wt levels of genome accumulation were seen in their corresponding protoplast infections (Figure 2B, C).

### The PRTE-DRTE interaction down-regulates viral RNA replication

**Figure 6:**
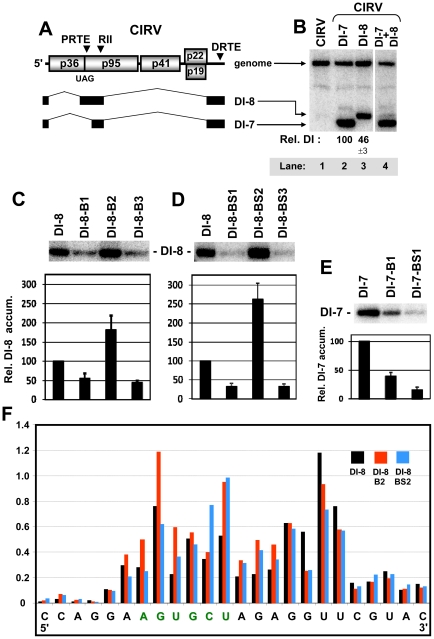
Assessing the role of the PRTE and DRTE in modulating viral RNA replication. (**A**) Schematic linear representation of the CIRV RNA genome and CIRV-derived DI-8 and DI-7 RNA replicons. The relative locations of the PRTE, RII, and DRTE are indicated above the genome. DI-8 and DI-7 are represented as a series of black boxes and thin intervening lines. The black boxes represent corresponding regions of the CIRV genome that are retained in the DI RNAs and thin lines represent regions that are absent. (**B**) Northern blot analysis and quantification of DI RNA accumulation when cotransfected with wt CIRV into plant protoplasts. DI-7 and DI-8 RNAs were analyzed 22 hr post-transfection and relative values below the lanes correspond to means (± standard error) from three independent experiments. (**C and D**) Northern blot analysis of DI-8 RNA accumulation when cotransfected with wt CIRV into plant protoplasts. Mutants DI-8-B1,-B2, -B3, -BS1, -BS2 and -BS3 contain the same substitutions as in genomic mutants B1, B2, B3, BS1, BS2, and BS3, respectively, as shown in Figure 2A. DI-8 RNAs were analyzed by Northern blot as described above. (**E**) Northern blot analysis of DI-7 RNA accumulation when cotransfected with wt CIRV in plant protoplasts. Mutants DI-7-B1, and -B2 contain the same substitutions as in genomic mutants B1 and B2, respectively, as shown in Figure 2A. (**F**) SHAPE analysis of the PRTE and its flanking sequence in wt DI-8 and mutant DI-8-B2 and DI-8-BS2. Relative reactivity of each nucleotide is plotted graphically, with larger values corresponding to increased flexibility. The PRTE is shown in green.

Based on the preceding results indicating a possible role for the long-range interaction in modulating additional viral processes and the fact that the interaction involved a previously characterized RNA replication element, RIV [27]–[29], we next investigated whether the interaction could influence viral RNA replication. For this assessment, replication needed to be uncoupled from RT, and this was accomplished using a small non-coding viral RNA replicon, DI-8, that contained the PRTE and DRTE (Figure 6A). Notably, in separate cotransfections with wt CIRV genome, DI-8 replicated less efficiently than DI-7 containing only the DRTE (Figure 6B, compare lanes 2 and 3) and DI-8 accumulated much less efficiently in cotransfections with DI-7 (Figure 6B, lane 4). Accordingly, part of the reduced fitness of DI-8 could be caused by an antagonistic effect of the PRTE-DRTE interaction on replication. To test this, the same disruptive and restorative substitutions that were present in the B- and BS-series mutants were introduced into DI-8. In coinoculations with wt CIRV genome into protoplasts, DI-8-B1 and DI-8-BS1 with substitutions in the DRTE showed reduced levels of accumulation, whereas DI-8-B2 and DI-8-BS2 with substitutions in the PRTE exhibited ∼1.8- and ∼2.6-fold enhanced levels of accumulation, respectively (Figure 6C, 6D). DI-8-B3 and DI-8-BS3 with the interaction restored showed levels similar to their DI-8-B1 and DI-8-BS1 counterparts (Figure 6C, 6D). The enhanced levels observed for DI-8-B2 and DI-8-BS2 are consistent with an inhibitory role for the interaction, while the results for DI-8-B1 and DI-8-BS1 suggest the opposite. One possible explanation for the latter result is that the DRTE substitutions in DI-8-B1 and DI-8-BS1 directly affected the replication function of RIV. To test this possibility, the same DRTE substitutions were introduced into DI-7, which lacks the PRTE and, thus, is independent of the interaction. Both DI-7-B1 and DI-7-BS1 showed accumulation defects similar to their DI-8 counterparts (Figure 6E), indicating that the reduced levels were not related to disruption of the interaction. Consequently, for DI-8-B2 and DI-8-BS2, it seems likely that the PRTE-DRTE interaction is antagonistic to replication, because modifications in the PRTE that destabilized it alleviated the inhibition. This notion is also consistent with the increased flexibility observed in the PRTE region in these DI-8 mutants (Figure 6F).

### An RNA switch in RIV modulates RT and RNA replication

**Figure 7:**
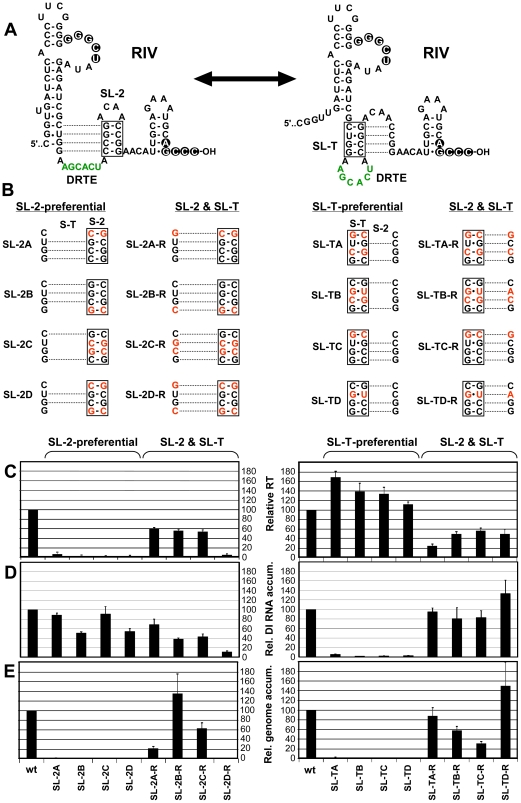
Effect of alternative conformations of RIV on RT and replication. (**A**) Schematic diagram of two possible RNA secondary structure conformations that RIV could adopt and influence the context of the DRTE. In the structure on the left, the replication-essential SL-2 is formed, whereas in the structure on the right the proposed SL-T is shown. Dotted lines denote the potential alternative base pairing in the two different structures and the double-headed arrow denotes the dynamic and mutually-exclusive relationship between the two conformations. (**B**) Substitutions in RIV (in red) predicted to influence formation of SL-2 and SL-T. “SL-2-preferential” mutants contain substitutions predicted to retain SL-2 formation and suppress SL-T formation. Conversely, “SL-T-preferential” mutants are predicted to do the opposite. The mutants in the two columns to the right of “SL-2-preferential” or “SL-T-preferential” are predicted to restore the balance of formation of “SL-2 & SL-T”, respectively, through additional substitutions. (**C**) Relative RT levels in wge for wt and mutant CIRV genomes containing substitutions shown in B. (**D**) Relative DI-7 accumulation levels in protoplasts for cotransfections of wt CIRV with wt DI-7 or DI-7 mutants containing the substitutions depicted in panel B. (**E**) Relative viral genome accumulation levels in protoplasts for wt and mutant CIRV genomes containing the substitutions shown in panel B.

Data from the preceding section suggested that the PRTE-DRTE interaction can modulate viral RNA replication. The known functions of RIV include mediating assembly of the viral replicase [28], acting as a promoter for minus-strand synthesis [35], and modulating minus-strand synthesis via formation of a pseudoknot between the 3'-end of the genome and a bulge in the large SL, termed the replication silencer element [27]. Due to the central role of RIV in viral RNA replication and the essential role for each of the three SLs in this region [36], [37] (Figure 7A, left panel), it seemed plausible that its interaction with the PRTE could alter its replication-related activities. Close examination of the structure of RIV revealed the possibility of an alternative central hairpin, termed SL-T, which would position the DRTE in its loop (Figure 7A, right panel). Interestingly, formation of either SL-T or replication-essential SL-2 would be mutually exclusive, and thus could provide a means to facilitate either RT via formation of SL-T or RNA replication via formation of SL-2 (Figure 7A). To test this hypothesis, sets of mutations were introduced into the CIRV genome that were predicted to preferentially stabilize one of the two SLs and, subsequently, additional substitutions were added to these mutants that were designed to restore a more wt-like thermodynamic balance of the two SLs (Figure 7B). In wge, genomes containing preferentially-stabilized SL-2 (SL-2A, -2B, -2C and -2D) did not yield efficient RT of p95, while additional substitutions geared to facilitate formation of both SLs (SL-2A-R, -2B-R, -2C-R and -2D-R) led to partial recovery of RT in three of the four mutants tested (Figure 7C, left panel). Conversely, genomes containing preferentially-stabilized SL-T (SL-TA, -TB, -TC and -TD) showed high levels of RT, and RT was less prominent when modifications designed to promote formation of both SLs were introduced in SL-TA-R, -TB-R, -TC-R and -TD-R (Figure 7C, right panel). Accordingly, the results support the concept that SL-2 is antagonistic to RT while SL-T is beneficial to this process.

Next, to assess the effects of the same mutations on RNA replication that was independent of RT, the same sets of mutations were introduced into DI-7 (Figure 7D). When cotransfected into protoplasts with wt CIRV genome, the DI-7 mutants containing preferentially-stabilized SL-2 accumulated between ∼50% to near wt levels and three of the four mutants with the additional changes designed to better balance the formation of the two SLs accumulated to ∼40-60% (Figure 7D, left panel). When DI-7 mutants containing preferentially-stabilized SL-T were tested, all were severely defective (Figure 7D, right panel), while engineering a closer balance between SL-2 and SL-T efficiently restored DI-7 accumulation in all mutants (Figure 7D, right panel). These results directly contrast those for RT, in that SL-2 formation was found to be compatible with replication while SL-T formation was inhibitory to this process.

When viral genomes harboring the modifications were assessed for replication in protoplasts, only those containing substitutions predicted to allow for the formation of both SL-T and SL-2 were able to accumulate (Figure 7E). Overall, these results are consistent with the concept that two alternative and mutually-exclusive conformations of RIV are required and involved in modulating RNA replication and RT, with SL-2 facilitating RNA replication and inhibiting RT, compared with SL-T enhancing RT and down-regulating replication.

**Figure 8:**
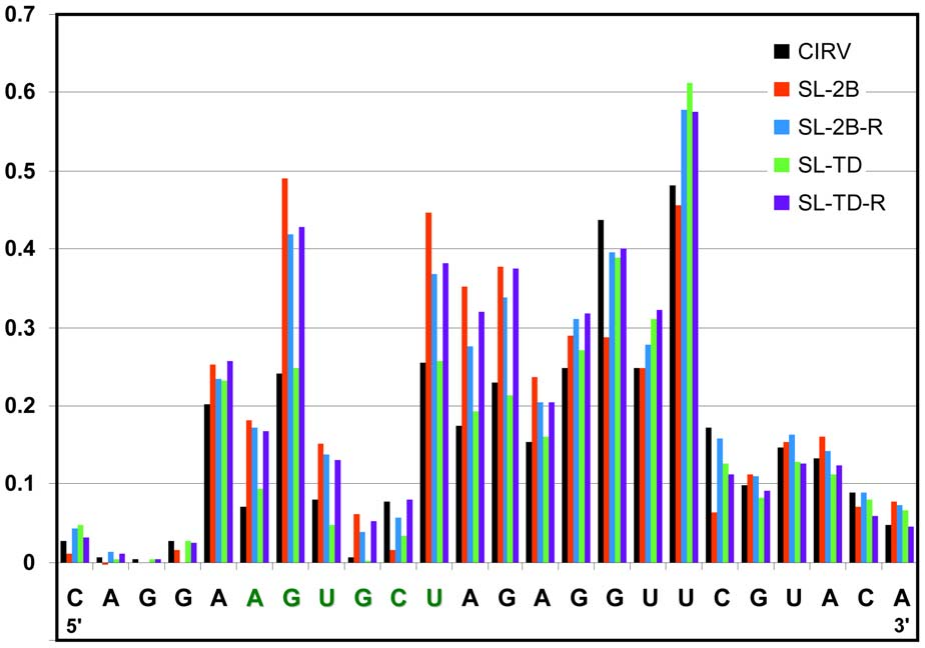
SHAPE analysis of the PRTE in viral genomes containing substitutions in RIV. Relative reactivity of each nucleotide is plotted graphically, with larger values corresponding to increased flexibility. The genomic mutants assayed are indicated in the key at the top right and correspond to those described in Figure 7B.

To test the possibility that regulation of RT in the cases above was linked to control over the formation of the PRTE-DRTE interaction, SHAPE analysis in the PRTE region of mutant genome pairs SL-2B, SL-2B-R and SL-TD, SL-TD-R was performed. The results indicated nucleotide reactivities that were in general agreement with the notion of PRTE-DRTE involvement (Figure 8). Specifically, preferential stabilization of SL-2 in mutant SL-2B increased PRTE reactivity relative to that for wt CIRV (Figure 8). This relative level of increase in reactivity was comparable to those seen for B1 and BS1 in Figure 3A and, consistent with the lack of RT observed for B1 and BS1 (Figure 2B), RT from mutant SL-2B was also eliminated (Figure 7C, left panel). Mutant SL-2B-R, which was designed for a better balance of the two SLs, showed a level of reactivity lower than its mutant SL-2B counterpart, but higher than wt CIRV (Figure 8), and this intermediate level of reactivity corresponded to partial recovery of RT (∼55%) for mutant SL-2B-R (Figure 7C, left panel). Preferential stabilization of SL-T in mutant SL-TD showed near wt levels of reactivity (Figure 8) and near wt levels of RT (Figure 7C, right panel). When the predicted stabilities of the two SLs were better balanced in mutant SL-TD-R, the level of reactivity increased and was similar to the intermediate level seen for mutant SL-2B-R (Figure 8) and, as observed for mutant SL-2B-R (Figure 7C, left panel), mutant SL-TD-R directed an intermediate level of RT (∼50%) (Figure 7C, right panel). The partial recoveries seen for mutants SL-2B-R and SL-TD-R suggests that the SL balances achieved were similar, but not identical, to that of wt CIRV, which is reasonable considering that these mutants contained multiple nucleotide substitutions. Importantly, overall, the structural data are consistent with the functional data and thus support the concept that alternative conformations of RIV are associated with differing levels of formation/stabilization of the PRTE-DRTE interaction.

### The UL-DL interaction facilitates RT

**Figure 9:**
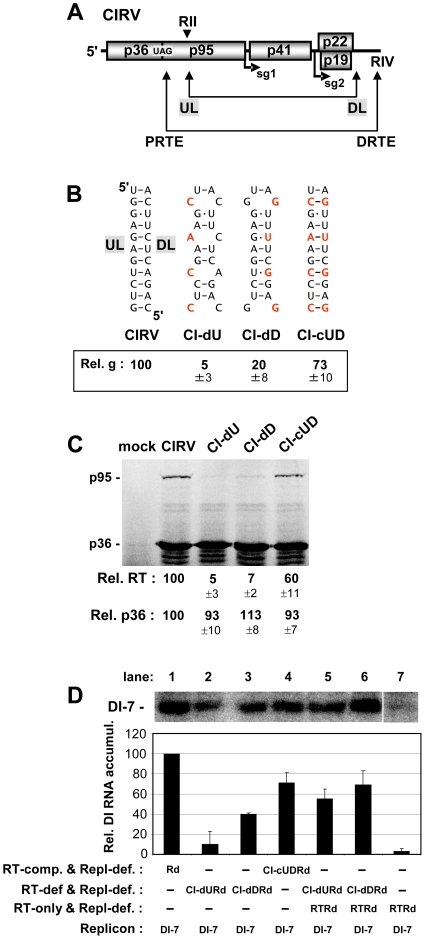
Analysis of the role of the UL-DL interaction in RT. (**A**) Schematic linear representation of the CIRV RNA genome showing the UL-DL and PRTE-DRTE interactions. The relative locations of RII and RIV are shown above and below the genome, respectively. (**B**) CIRV genomic mutants with compensatory mutations in the UL-DL interaction. Substitutions are shown in red. Previous results of viral genome accumulation levels from protoplast transfections are shown in the box below [24]. (**C**) In vitro translation in wge of CIRV genomes containing mutations shown in panel B. (**D**) Analysis and quantification of DI-7 accumulation by Northern blot analysis (top) 22 hr post-cotransfection with non-replicating CIRV genomes in plant protoplasts. The CIRV genomes cotransfected with DI-7 are indicated below each bar in the graph.

Our inability to demonstrate RT using a dual luciferase reporter mRNA with relevant viral regulatory regions (data not shown) suggested to us that the viral genomic context may be important for RT activity. Indeed, the multiple functional long-range interactions that occur in tombusviruses suggest that proper global folding of the viral genome is critical for proper operation of many viral processes [24]. Since the PRTE was found to interact with RIV, and RIV communicates with RII via a long-range UL-DL interaction [24] (Figure 9A), we wondered whether the latter replication-related interaction could also influence RT. The UL-DL interaction is important for CIRV genome replication, as shown previously [24] (Figure 9B). However, previous analysis of CIRV genomes with disruptions and restoration of the UL-DL interaction did reveal a role for this interaction in regulating the efficiency of p36 translation [24]. Similarly, we found that p36 levels in vitro were not notably altered by disruption of the UL-DL interaction, however RT was considerably reduced when this interaction was destabilized (Figure 9C).

To determine if a similar defect also occurred in vivo, an approach akin to that described in Figure 4 was used. Replication-defective versions of the UL-DL compensatory mutants were generated and tested for their ability to amplify DI-7. Disruption of the interaction in CI-dURd and CI-dDRd led to reduced levels of DI-7 accumulation, while restoring the interaction in CI-dUDRd led to partial recovery (Figure 9D, lanes 2, 3 and 4). DI-7 replication was enhanced ∼5.5- and ∼1.75-fold when RTRd (providing p95) was cotransfected with CI-dURd or CI-dDRd, respectively, consistent with RT defects in the latter two (Figure 9D, lanes 5 and 6). Collectively, the results support a role for the long-range UL-DL interaction in facilitating RT in vitro and in vivo.

### Long-range RNA-RNA interactions are predicted for all genera of Tombusviridae that use RT to express their RdRp

**Figure 10:**
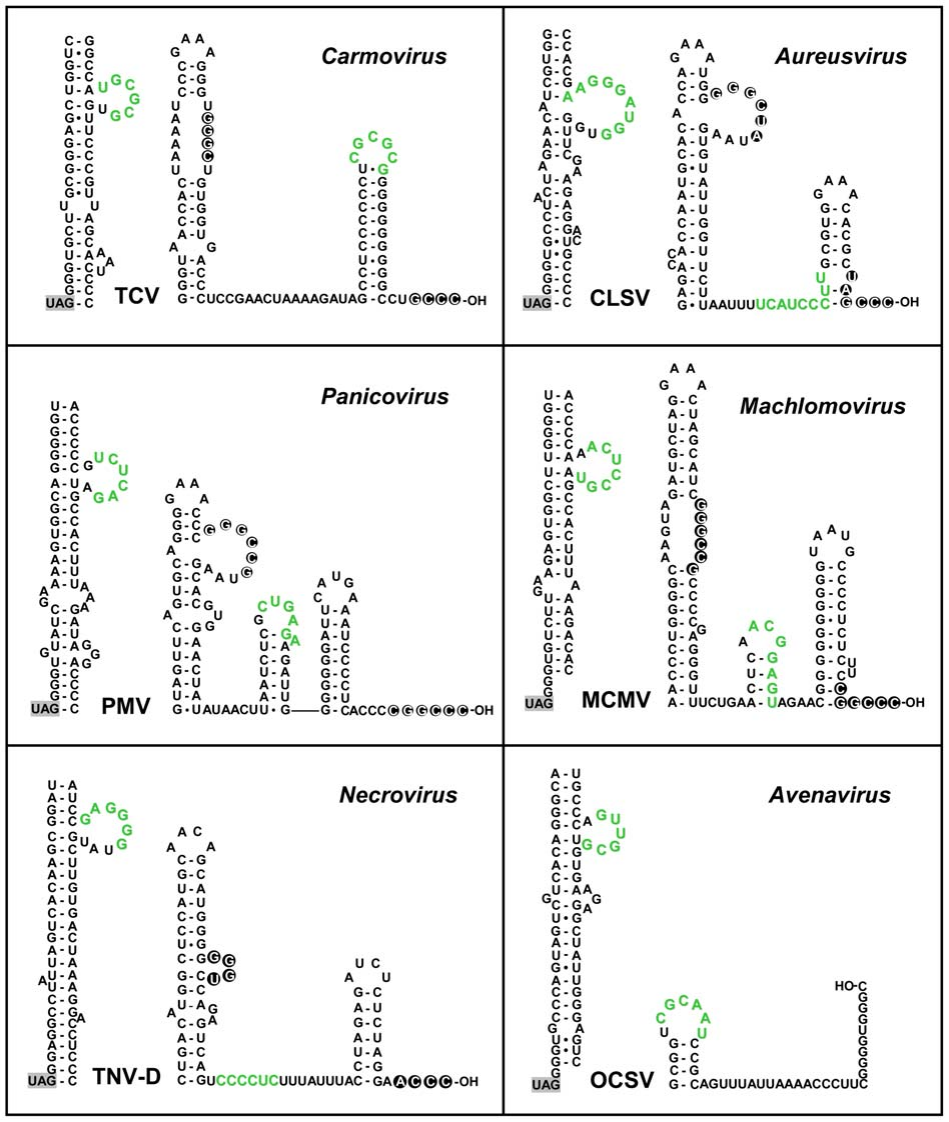
Conservation of potential PRTE-DRTE-like interactions in members of the family Tombusviridae. Predicted RNA secondary structures for the sequences 3'-proximal to the RT stop codons (on left in each panel) and the 3'-terminal sequences (right) in selected species of genera in Tombusviridae. Sequence segments that could potentially form PRTE-DRTE-like interactions are shown in green and the RT stop codon is highlighted in grey. The virus acronyms are: TCV, Turnip crinkle virus; CLSV, Cucumber leaf spot virus; PMV, Panicum mosaic virus; MCMV, Maize chlorotic mottle virus; TNV-D, Tobacco necrosis virus D; OCSV, Oat chlorotic stunt virus.

Having established a role for the PRTE-DRTE interaction in the tombusvirus CIRV, we wondered whether this phenomenon also extended to other genera in Tombusviridae. Sequence and structural analysis of the six other genera that utilize RT to express their RdRp revealed the potential for formation of extended SL structures just 3' to their RT sites that contained bulges with sequences that were complementary to 3'-terminal sequences in their viral genomes. Examples of these sequences and predicted structures for the best-studied members of these six genera are presented in Figure 10 [11]. Interestingly, although the SLs with the PRTEs in a bulge were similar in general structure, the relative locations of the DRTEs at the 3' ends varied. For example, in Turnip crinkle virus (TCV), the DRTE is predicted to reside in the apical loop of the 3'-terminal SL, termed Pr, which is the core promoter for minus-strand synthesis [38] (Figure 10). However, for other carmoviruses, like Saguaro cactus virus, the DRTE is instead located in the apical loop of the larger internal SL commonly termed the replication silencer element (data not shown) [39]. In other viruses, such as Panicum mosaic virus (PMV), Maize chlorotic mosaic virus (MCMV) and Oat chlorotic stunt virus (OCSV), the DRTE is located in predicted smaller SLs, while in Cucumber leafspot virus (CLSV) and Tobacco necrosis virus-D (TNV-D) the DRTE is predicted to reside in an intervening ssRNA region between internal and 3'-terminal SLs (Figure 10). Based on these observations, it seems likely that long-range RNA-RNA interactions similar to that in CIRV also exist and operate to facilitate RT in other genera of Tombusviridae.

**Figure 11:**
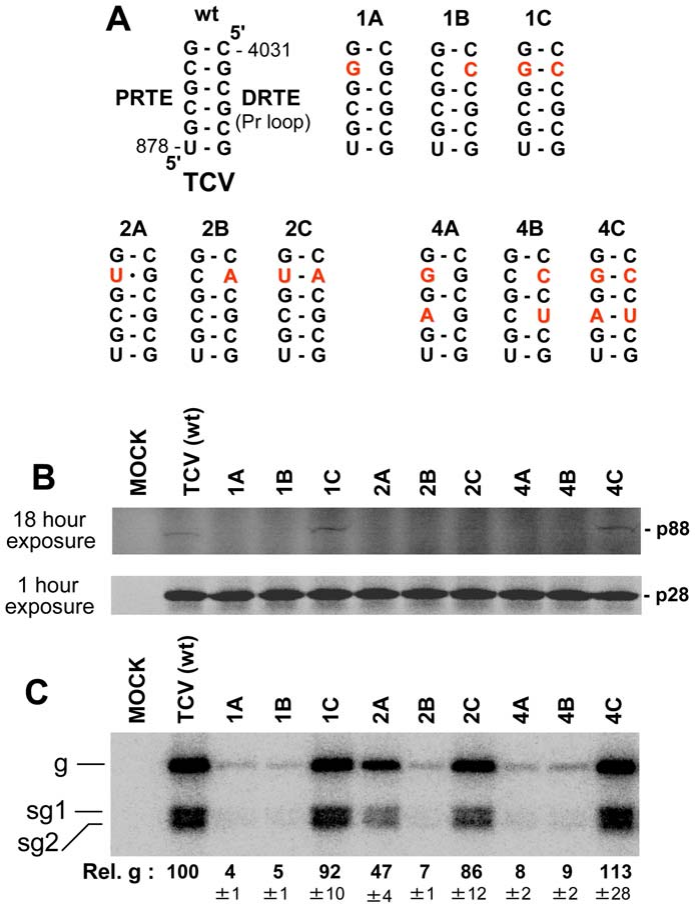
Analysis of the proposed PRTE-DRTE in TCV. (**A**) TCV genomic mutants with compensatory mutations in the PRTE-DRTE interaction. Mutated residues are shown in red. (**B**) In vitro translation in wge of TCV genomes containing mutations shown in panel A. The same gel was exposed for different times, as indicated, in order to better visualize bands corresponding to p88 and p28. (**C**) Northern blot analysis of wt and mutant TCV genomes in protoplasts and quantification of plus-strand viral genome accumulation.

To provide some experimental support for this proposal, we selected one virus genus to test the concept. The carmovirus TCV was selected as: carmovirus represents the largest genera in Tombusviridae; previous studies attempted, unsuccessfully, to define the RT elements in TCV [40], [41] and; the 3'-terminus of TCV is well-defined structurally and functionally [42]–[47]. Accordingly, three different sets of compensatory mutants were generated in which the complementarity between the predicted PRTE and DRTE was reduced and then restored in the TCV genome (Figure 11A). In wge, very low but reproducible and detectable levels for RT were observed from the wt TCV genome and from mutants 1C, 2C and 4C, where complementarity was restored (Figure 11B). This correlation between maintenance of the PRTE-DRTE pairing potential and RT was also supported by protoplast transfections, where near-wt levels of replication of the TCV genome were observed only for mutants 1C, 2C and 4C (Figure 11C). Based on these results, it appears that the carmovirus TCV also utilizes a long-range interaction to facilitate RT and this finding bolsters the concept that all members of Tombusviridae that express their RdRps by RT also utilize a similar mechanism. Indeed, comparable studies with the necrovirus TNV-D also indicate a role for the proposed long-range interaction depicted in Figure 10 in mediating RT (unpublished data, B. L. Nicholson, K. A. White).

## Discussion

Diverse arrays of viruses utilize translational RT in their gene expression strategy, including the majority of the members of the large virus family Tombusviridae [7], [8]. Here we provide evidence that the tombusvirus CIRV utilizes two sets of long-range RNA-RNA interactions and an RNA switch that are functionally linked to RNA replication elements to modulate and coordinate RT and viral genome replication. Some features of the proposed regulatory mechanisms also likely apply to other members of Tombusviridae.

### Structural aspects of the RNA elements involved in RT

**Figure 12:**
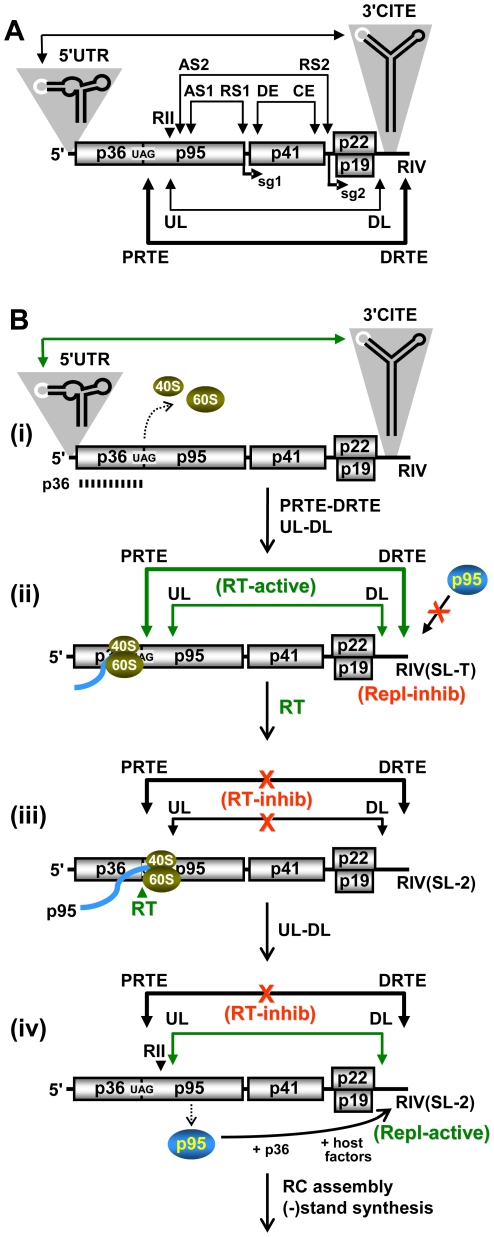
Proposed RNA-based regulatory network modulating RT and genome replication in CIRV. (**A**) Schematic linear representation of the CIRV RNA genome showing all defined functional long-range RNA-RNA interactions in tombusviruses. The 5'UTR-3'CITE interaction facilitates efficient initiation of translation [18]; the AS1-RS1 interaction mediates sg mRNA1 transcription [22]; the AS2-RS2 and DE-CE interactions mediate sg mRNA2 transcription [21], [23]; the UL-DL interaction facilitates replicase complex assembly [24] and RT (this study); and the PRTE-DRTE interaction mediates RT (this study). (**B**) Events leading to the translation of p36 and p95 and subsequent RNA replicase complex assembly. **(i)** The 5'UTR-3'CITE interaction mediates efficient initiation of translation that allows for production and accumulation of p36. **(ii)** Formation of the PRTE-DRTE and UL-DL interactions makes it possible for elongating ribosomes to readthrough the p36 stop codon. Also, since the PRTE-DRTE interaction involves the SL-T-containing conformation of RIV, which inhibits the replication function of RIV, any p95 generated and provided in trans will not be utilized. **(iii)** Following RT, further 3'-movement of the translating ribosome leads to disruption of both the PRTE-DRTE and UL-DL interactions, which down-regulates RT and p95 production and allows RIV to adopt its SL-2-containing replication-active conformation. **(iv)** Reformation of the UL-DL interaction with the replication-active RIV generates a functional RII-RIV RNA platform that is used by the p95 translated in cis, along with p36 and host factors, to assemble into an active viral RNA replicase complex (RC) that carries out minus-strand synthesis.

We have identified and characterized SL-PRTE as a structure located 3'-proximal to the CIRV RT site that is necessary for RT-based translation of p95 (Figure 2). However, on its own, this structure was unable to direct efficient RT and required a second RNA element for function, the DRTE, located in RIV at the 3'-end of the viral genome. A base-pairing interaction spanning a distance of ∼3.5 kb between the PRTE and DRTE was necessary for efficient RT and, as such, it represents the sixth functional long-range (*i.e.* ≥ ∼1 kb) RNA-RNA interaction to be identified in tombusviruses (Figure 12A). Interestingly, this interaction provides a direct physical link between the SL-PRTE structure and the functionally-characterized RNA replication element, RIV. The DRTE in RIV is highly integrated with RIV at different structural levels. First, it represents a “linear” intervening sequence between SL-2 and the replication silencer element SL-3 (Figure 2A). As such, it could serve as a necessary spacer sequence between these two secondary structures and, indeed, deletion of this sequence from DI-7 results in severely reduced accumulation of this replicon (Figure S4). Additionally, the sequence identity of the DRTE also contributes to replication functions, as substitutions within it compromise DI-7 accumulation (Figure 6E and Figure S4). Another level of integration of the DRTE with RIV is at the secondary structure level, where our mutational analysis supports the formation of an alternative SL in RIV, SL-T, which positions the DRTE in its loop (Figure 7A). Such a structure, could (i) form before the long-range interaction and facilitate nucleation of the PRTE-DRTE interaction or/and (ii) form after the long-range interaction and provide a helix for coaxial stacking with the base-paired PRTE-DRTE. The former would facilitate more efficient formation of the interaction, while the latter would act to further stabilize the interaction. Regardless of the mode of action, the formation of SL-T would prevent SL-2 from arising, because the two structures are mutually exclusive (Figure 7A), and since SL-T facilitates primarily RT while SL-2 mediates principally RNA replication, the inter-conversion of the two structures could provide a means to regulate these two processes (*i.e.* an RNA switch). Such RNA switches have been implicated in the regulation of a variety of processes in viruses [48] such as Dengue virus [49], [50], coronaviruses [51], HIV [52] and satellite-C RNA of TCV [53], [54].

In addition to the long-range interaction and RNA switch, our study uncovered an additional level of control. Efficient RT also requires the UL-DL long-distance interaction that is important for RNA replication. This represents a second link between RT and RNA replication. The specific role of the UL-DL in facilitating RT will be investigated in detail in subsequent studies, however its general proximity to the SL-PRTE and RIV suggests that it could act to bring together these RNA elements in the folded genome (Figure 12A). This concept, that proper global viral genome folding is important for RT, is also consistent with our inability to demonstrate PRTE-DRTE-dependent RT using the heterologous context of a dual luciferase reporter mRNA. Accordingly, two distinct long-range interactions are necessary for RT, the PRTE-DRTE that forms a critical tertiary interaction proximal to the RT site and the UL-DL which likely plays a less direct role by mediating functionally-important global folding of the viral genome. Additionally, the 5'UTR-3'CITE interaction that mediates efficient initiation of translation may also assist in RT, albeit to a lesser extent, as there was a drop in relative RT to ∼37% that of wt when the 3'CITE was deleted (Figure 1C).

### Regulatory aspects of the RNA elements involved in RT

The production of RdRp is a critical step in the replication cycle of CIRV and other plus-strand RNA viruses [55]. Accordingly, viruses have developed strategies to produce RdRp at appropriate times and in the required amounts. In tombusviruses, the production of RdRp is coordinated with the assembly of viral replication complexes and subsequent minus-strand synthesis of genomes [11]. Exactly how this process is successfully orchestrated remains to be determined, however our results provide some insights into how these events could unfold.

An obvious question regarding RT in CIRV is: why is an essential RNA element for RT located at the 3'-end of the virus genome? Several possible explanations, which are not necessarily mutually exclusive, come to mind. First, the terminal location of the DRTE may provide a quality control function during virus reproduction. That is, little or no RdRp would be produced from viral genomes that are missing their 3'-termini due to incomplete replication or ribonuclease attack. Such a safety check for genome completeness would be very sensitive for cases like TCV, where the DRTE is located at the extreme end of the viral genome (Figure 10).

A second possible role for the distal location of the DRTE could be related to promoting cis-preferential replication, where the RdRp acts on the genome from which it is translated. This phenomenon has been reported for Red clover necrotic mosaic virus (RCNMV, genus Dianthovirus, family Tombusviridae) [56] and, likewise, we found that RT-defective B2 and BS2 CIRV genomes could not be rescued by providing p95 in trans (Figure S5). This type of cis-coupling may be important for efficient replication of invading genomes at low multiplicity of infection. The strategy would also ensure that RT-defective genomes would not be amplified by RdRp provided in trans from RT-competent genomes. Moreover, cis-preferential replication would facilitate the co-evolution of the RdRp with its cognate genome. Considering these potential benefits, how then could cis-preferential replication be mediated in CIRV by RT? A possible answer lies in the link between RT and RIV. If the replication function of RIV is inhibited when bound to SL-PRTE, as suggested by our results (Figure 6 and 7), then an RdRp generated from another genome would be less able to utilize it (Figure 12B (ii)). However, the PRTE-DRTE interaction would stimulate RT in cis, and subsequent translation of p95 from the genome would separate the SL-PRTE-RIV interaction (Figure 12B (iii)). This disruption by translating ribosomes would then allow for conversion of RIV to its replication-active form at the same time that p95 is being synthesized and is in proximity (Figure 12B (iv)). Accordingly, in this scenario, the PRTE-DRTE interaction would provide the physical link that synchronizes RT with RNA replication for individual viral genomes.

A third reason for the 3'-terminal location of the DRTE, which is related mechanistically to that just described, could be to coordinate translation with RNA replication. Initially, in the RT mode, the inactivation of the replication function of RIV would help to prevent viral replicase initiating RNA synthesis in the 3'-to-5' direction on the genome during translation of the RdRp (Figure 12B (ii)), which would presumably lead to incomplete minus-strand production due to ribosome displacement of the replicase. Conversely, the subsequent dissociation of RIV from SL-PRTE by translation of p95 would cause RT to be down-regulated, thereby providing an opportunity for the replicase to copy the genome free of ribosome traffic (Figure 12B (iv)). Interestingly, the UL-DL interaction required for RT would also be disrupted by the translation of p95 (Figure 12B (iii)), and this same interaction is required for subsequent assembly of the viral RNA replicase complex [24]. Accordingly, the UL-DL interaction would have to reform following p95 synthesis, while the PRTE-DRTE interaction would need to be inhibited to prevent further RT (Figure 12B (iv)). The latter step could be facilitated by a yet to be determined p95-mediated event that prevents the DRTE in RIV from re-pairing with the PRTE. Such coordination of viral translation and replication could be important at early or intermediate stages in the infection when protective membrane spherules associated with tombusvirus RNA replication centers are not fully formed [26].

Overall, the combination of multiple regulatory elements and the structural link of RT with replication elements likely provide the virus with benefits that include quality control of viral genomes, cis-preferential replication, and coordination of translation with RNA replication. Precisely how the PRTE and DRTE mediate RT remains unknown, but possibilities include the interaction forming a higher-order RNA structure that promotes ribosome stalling and/or the recruitment of a factor(s) that inhibits translation release factor function or promotes suppressor tRNA utilization. Further studies are ongoing to investigate such prospects.

### Comparative and evolutionary aspects of the RNA elements involved in RT

This is the first report demonstrating the requirement for a long-range RNA-RNA interaction (spanning ∼3.5 kb) for RT in a plus-strand virus. However, based on sequence analysis (Figure 10) and experimental data (Figure 11 and unpublished data), PRTE-DRTE-like interactions appear to also exist in other viruses in Tombusviridae. Accordingly, some aspects of the mechanisms proposed for CIRV likely also apply to these viruses, however the details may differ. For example, in TCV, the 3'-terminal Pr SL that contains the DRTE is the core promoter [38], therefore, for this virus, direct RdRp binding may be the regulatory mechanism shutting down RT and allowing minus-strand synthesis to proceed unimpeded. Thus, although the presence of PRTE-DRTE-like interactions provide the general basis for a common family-wide mechanism, each genus has probably evolved unique aspects dictated by its particular genomic context and host-specific selection pressures.

In terms of the relative timing of appearance of the long-range interactions during virus evolution, we envision the initial RT signal as being local. Subsequently, through random events and “testing” of various alternative long-range interactions, the original local structure would then morph into one incorporating a distal element, due to associated fitness gains. The long-range RNA-RNA interaction proposed for RT of the coat protein in Barley yellow dwarf virus (BYDV) [57] suggests that this phenomenon may also extend to different types of proteins and to viruses outside of the family Tombusviridae. Indeed, we have identified potential PRTE-DRTE-like long-range interactions that could mediate RT of RdRp in both pelarspoviruses and umbraviruses (K. A. White, unpublished data). These types of long-range interactions contrast the shorter-range interactions that have been described in retroviruses (spanning 8 nt) [58], [59] and alphaviruses (spanning ∼100-150 nt) [8]. The retroviral interaction in Moloney murine leukemia virus (MMLV) that leads to the production of a gag-pol fusion protein involves a local pseudoknot and a sequence surrounding the retrovirus stop codon (UAGGGGUGU) [58], [59]. This sequence is similar to those in many members of Tombusviridae (Figure 10) [7] and the PRTE-DRTE interaction involving a bulge in SL-PRTE is somewhat structurally similar to the pseudoknot in MMLV. Accordingly, these RT elements from different virus classes may also share some mechanistic features. In contrast, the shorter-range interactions required for RT in alphaviruses form simple stem structures and involve a different stop codon and context, UGAC [8], suggesting a different mode of action.

Interestingly, functional long-distance RNA-RNA interactions spanning ∼4 kb and ∼2.6 kb, respectively, have been described for the frameshifting leading to RdRp production in the Luteovirus BYDV (family Luteoviridae) [15] and the Dianthovirus RCNMV (family Tombusviridae) [16]. Structurally, there are some very striking similarities in the proximal and distal RNA structures involved in these interactions and those described herein for RT. The proximal SL for both BYDV and RCNMV, similar to the SL-PRTEs in Tombusviridae, contain a bulge in the lower 3'-half of a long stem that pairs with a distal RNA element. Also, akin to some of the DRTEs (Figure 10), the distal frameshifting elements reside in loop regions of stable SL structures [15], [16]. Conversely, both distal frameshifting elements are located away from the 3'-terminus harboring the core promoter for minus-strand synthesis [15], [16], suggesting a modular rather than integrated context with respect to replication elements. However, although their relative positions suggest differently, it is possible that these distal frameshifting elements may also modulate RNA replication [15], [16], but such prospects remain to be investigated. Nonetheless, the resemblance between the structures and interactions of the distal and proximal elements for RT and frameshifting is striking and suggests a possible common ancestry as well as potential mechanistic similarities between these distinct recoding processes.

## Materials and Methods

### Plasmid construction

The full-length infectious clone of CIRV has been described previously [17] and all CIRV mutants in this study were derived from this construct. Using standard recombinant DNA cloning techniques [60], an MluI restriction site was engineered at nt position 1314–1319 (that maintained the wt amino acid sequence) in order to facilitate PCR-based oligonucleotide-mediated mutagenesis of the CIRV genome. In vitro transcripts from the CIRV genomic construct containing the MluI site mediated efficient RT in vitro and replicated in protoplasts at levels comparable to the unmodified genome (Figure S6). Accordingly, in this study the former is referred to as the wt CIRV genome. PCR-derived regions containing the designed modifications that were introduced into constructs were sequenced completely in order to verify that only the desired changes were present. All mutations are shown schematically in the figures in this study.

The construction of DI-7 was based on the sequence of a naturally-occurring CIRV DI RNA [17]. To make DI-7, three regions of the CIRV genome were amplified by PCR: (i) the 5'-terminal 150 nt, (ii) an internal region consisting of nt 1344–1479, and (iii) the 3'-terminal 371 nt. These regions were joined using a KpnI site between regions (i) and (ii) and a SalI site between regions (ii) and (iii), and ligated into pUC19 using an EcoRI site upstream of (i) and a HindIII site downstream of (iii). Additionally, a T7 promoter was introduced at the 5'-end of the construct and a SmaI site was inserted at the 3'-end allowing for generation of an authentic 3'-terminus. DI-8 was made from DI-7 by replacing the internally-derived segment with nts 1067-1479 of the CIRV genome. The entire constructs were sequenced completely to confirm that the regions in the DI RNAs were correct. Mutants of DI-7 and DI-8 were made by standard PCR-based mutagenesis and all constructs were confirmed by sequencing.

Construction of TCV mutants was performed using the genomic construct T1D1 [61] employing standard PCR-based mutagenesis. PCR-derived regions containing the designed modifications that were introduced into constructs were sequenced completely in order to verify that only the desired changes were present. All mutations are shown schematically in Figure 11.

### Computer-aided analysis of RNA

The genome sequences of AMCV (X62493), CBLV (NC_004725), CIRV (NC_003500), CuNV (NC_001469), CymRSV (NC_003532), GALV (AY830918), PLV (NC_004723), PNSV (AJ607402), TBSVc (NC_001554), TBSVnf (AY579432), TBSVp (U80935), and TBSVs (AJ249740) were obtained from the NCBI GenBank database. RNA secondary structures were predicted at 37°C using Mfold version 3.5 [30], [31].

### In vitro transcription, protoplast transfection, and viral RNA analysis

Uncapped RNA transcripts were generated from DNA plasmids linearized with SmaI (viral genomic and DI RNAs) or PCR templates (small RNAs used for EMSA studies) with a T7 promoter as described previously [62] using AmpliScribe T7-Flash transcription kits (Epicenter Technologies). Capped transcripts were generated from DNA plasmids linearized with SmaI using AmpliCap T7 High-Yield Message Maker kits (Epicenter Technologies). Cucumber cotyledon protoplast preparation, RNA transfection, and total nucleic acid extraction were performed as outlined previously [22]. Briefly, 3×10^5^ cucumber protoplasts were transfected with RNA transcripts (3 µg for genomic CIRV RNA; 5 µg for non-replicating genomic RNA; 2 µg of non-replicating RTRd RNA; 1 µg for DI-7 or DI-8; 5 µg for genomic TCV RNA) and incubated at either 22°C (cotransfection with DI RNAs only, as the DI RNAs are temperature sensitive) or 26°C (all other transfections) for 22 hr. A minimum of three trials were conducted for each experiment. Total nucleic acid was isolated post-incubation and Northern blot analyses were conducted to detect plus-strand viral RNAs as described previously [22]. Nucleic acids were separated in 1.4% agarose gels and uniform loading for all samples was confirmed prior to transfer via staining the gels with ethidium bromide. ^32^P-labeled DNA probes complementary to the 3'UTR of the genomes were used for viral detection and bands on membranes were quantified using a PharosFX Plus Molecular Imager (BioRad) and QuantityOne software (BioRad).

### In vitro translation

Translation of sub-saturating concentrations of viral in vitro transcripts (0.5 pmol) in the presence of ^35^S-Met using nuclease-treated wheat germ extract (Promega) was performed as described previously [18], except that 100 mM KOAc was used to optimize the level of readthrough. Unless specified (as in Figure 1C), all in vitro-generated transcripts analyzed were uncapped. In vitro translation products were then separated by SDS-10%PAGE and quantified by radioanalytical scanning using a PharosFX Plus Molecular Imager (BioRad) and QuantityOne software (BioRad).

### RNA-RNA gel shift assay

RNA-RNA electrophoretic mobility shift assays were performed as described previously [39], with some modifications. Briefly, unlabelled RNAs were used and equimolar ratios of RIV and SL-PRTE RNAs (16 pmol each) were incubated for 30 min at 25°C in RNA binding buffer (5 mM HEPES at pH 7.8, 100 mM KCl, 6 mM MgCl_2_, 3.8% glycerol) in a total volume of 8 µL. Samples were subsequently cooled at 4°C for 10 min, mixed with loading buffer (50% glycerol, 50% RNA binding buffer), and separated at 4°C in a nondenaturing 15% polyacrylamide gel containing 6 mM MgCl_2_ using 1X TBE running buffer containing 6 mM MgCl_2_. Gels were then stained with ethidium bromide to visualize bands and the negative image is shown in Figure 3C.

### SHAPE Analysis

SHAPE analysis was performed on in vitro transcribed CIRV genomes and DI RNAs essentially as described previously [63]. Briefly, in a final volume of 60 µL, CIRV genomic RNA transcripts (4 pmol) with 30 µL of 1X TE were refolded by heating at 95°C for 5 min and incubated on ice for 2 min. 30 µL of 3.3X folding buffer, (333 mM NaCl, 16.5 mM MgCl_2_, 333 mM HEPES pH 8.0) was added, and samples were incubated at 37°C for 30 min. 45 µL of refolded RNA was treated with 5 µL of 50 mM 1-methyl-7-nitroisatoic anhydride (1M7) in dimethylsulfoxide (DMSO) at 37°C for 4 min. As a negative control, an additional 45 µL of refolded RNA was treated with 5 µL of DMSO under the same conditions. RNA was recovered by ethanol precipitation (with 200 mM NaCl, 2 mM EDTA, and 40 µg of glycogen) and the precipitated RNA was resuspended in 50 µL of 0.5X TE. To achieve detection of 2’-O-adducts by primer extension, a procedure modified from Wilkinson et al. [64] was used. Specifically, 6 µL of fluorescently-labeled primers were added to 20 µL of 1M7-treated (4 µM WellRED D4) and untreated (3 µM WellRED D3) RNAs and subsequently incubated at 65°C for 5 min and 37°C for 5 min. To carry out primer extension, enzyme mix (6 µL containing 250 mM KCl, 10 mM MgCl_2_, 167 mM Tris-HCl pH 8.3, 1.67 mM each dATP, dCTP, dITP, dTTP placed at 50°C for 2 min) and SuperScript III (1.5 µL, 300 units) were added and extension was carried out for 30 min at 50°C. For sequencing purposes, 20 µL of refolded RNA was combined with primer (6 µL containing 4 µM WellRED D2 or 4 µM IRDye 800), enzyme mix (6 µL), ddGTP (1 µL; 0.25 mM) or ddTTP (1 µL; 10 mM), respectively, SuperScript III (1.5 µL, 300 U) and primer extension was carried out. One set of primers were used that are complementary to CIRV positions 1320-1339 (5’-CCACAACCTACCAAAGGAGC). To terminate reactions, 3 M NaOAc pH 5.2 (4 µL) was added to each reaction and all reactions were combined and precipitated at -80°C for 15 min with ethanol (320 µL) and glycogen (2 µL). Pellets were washed twice with 70% ethanol, dried for 10 min at 37°C, and resuspended in 45 µL of deionized formamide. Raw fluorescence intensity versus elution time profiles were determined as described previously [65] and each nucleotide reactivity score was plotted graphically. Raw fluorescence intensity versus elution time profiles were analyzed using ShapeFinder [66]. The average for the top 10 peak intensities was calculated and all reactivities were divided by this average. This normalization procedure placed all absolute reactivities on a scale of 0 to approximately 1.5 and the average relative reactivities from two experiments were plotted graphically.

## Supporting Information

Figure S1 **SHAPE analysis of SL-PRTE RNA.** SHAPE analysis was performed on the wt CIRV genome and the results were mapped onto the mfold-predicted RNA structure for SL-PRTE. Relative reactivity of each residue is indicated by the color-coded key, with higher values corresponding to increased flexibility.
(TIF)

Figure S2 **Comparative RNA sequence and secondary structure analysis of SL-PRTE and the PRTE-DRTE interaction. (A)** The CIRV SL-PRTE secondary structure and **(B)** CIRV PRTE-DRTE interaction are shown along with sequence variations found in other tombusviruses. Nucleotides involved in the PRTE-DRTE interaction are shown in green and the p36 stop codon is in bold and underlined. Gray shading indicates CIRV nucleotides that are substituted by different nucleotides in other tombusviruses, with the corresponding identity of the virus indicated in brackets. The virus acronyms are as follows: AMCV, Artichoke mottled crinkle virus: CBLV, Cucumber Bulgarian latent virus; CuNV, Cucumber necrosis virus; CymRSV, Cymbidium ring spot virus; GALV, Grapevine Algerian latent virus; LNV, Lisianthus necrosis virus; PLV, Pear latent virus; PNSV, Pelargonium necrotic spot virus; TBSVc, Tomato bushy stunt virus (cherry isolate); TBSVnf, Tomato bushy stunt virus (nipple fruit isolate); TBSVp, Tomato bushy stunt virus (pepper isolate); TBSVst, Tomato bushy stunt virus (statice isolate).
(TIF)

Figure S3 **RdRp activity is required to complement RT-deficient mutants.** Northern blot analysis and quantification of DI-7 accumulation when cotransfected with non-replicating CIRV genomes in plant protoplasts. The CIRV genomes cotransfected with DI-7 are indicated below each bar in the graph. The mutants tested are described in Figure 4, except for mutant RTRdm, which contains a GDD-to-AAA triple codon substitution in its p95 GDD RdRp motif. DI-7 was analyzed 22 hr post-transfection of plant protoplasts. Northern blot analysis (top) was used to quantify the relative levels of DI-7 accumulation shown in the bar graph (± standard error) that was derived from three independent experiments.
(TIF)

Figure S4 **Importance of the DRTE for DI-7 replication. (A)** Predicted secondary structure for RIV with the DRTE shown in green. The substitutions and deletion made in the DRTE are shown below the structure. **(B)** Northern blot analysis and quantification of wt and mutant DI-7 RNA accumulation when cotransfected with wt CIRV in plant protoplasts. DI-7 RNA levels were analyzed and measured by Northern blot analysis 22 hr post-transfection of plant protoplasts. The relative values shown in the bar graph below the lanes correspond to means (± standard error) from three independent experiments.
(TIF)

Figure S5 **RT-defective genomes are not rescued by p95 supplied in trans.** Northern blot analysis and quantification of CIRV genomes analyzed 22 hr post-transfection of plant protoplasts. The identities of the viral genomes are shown above the lanes. Cotransfection of CIRV genome mutants B2 or BS2 with non-replicating RTRd (providing p95) did not lead to rescue of genome replication. The relative values for genome accumulation shown below the lanes correspond to means (± standard error) from three independent experiments.
(TIF)

Figure S6 **Analysis of CIRV genome containing an engineered MluI restriction. (A)** SDS-10%PAGE analysis of p36 stop codon RT from wt CIRV and CIRV containing an MluI restriction site (CIRV MluI). The p95:p36 ratio was determined for each lane, and the relative RT percentages below each lane correspond to means (± standard error) from three independent experiments. **(B)** Northern blot analysis and quantification of plus-strand accumulation of viral RNAs from CIRV WT and CIRV MluI transfections of plant protoplasts. The positions of the genomic (g) and subgenomic mRNAs (sg1 and sg2) are indicated to the right of the blot. Viral RNAs were analyzed 22 hr post-transfection and the relative values for genomic accumulation below the lanes correspond to means (± standard error) from three independent experiments.
(TIF)
