# Supplementary figures and images for: Multifaceted Regulation of Translational Readthrough by RNA Replication Elements in a Tombusvirus
Source: PLoS Pathog. 2011 Dec 8;7(12):e1002423. doi: 10.1371/journal.ppat.1002423 (PMC3234231; doi:10.1371/journal.ppat.1002423)

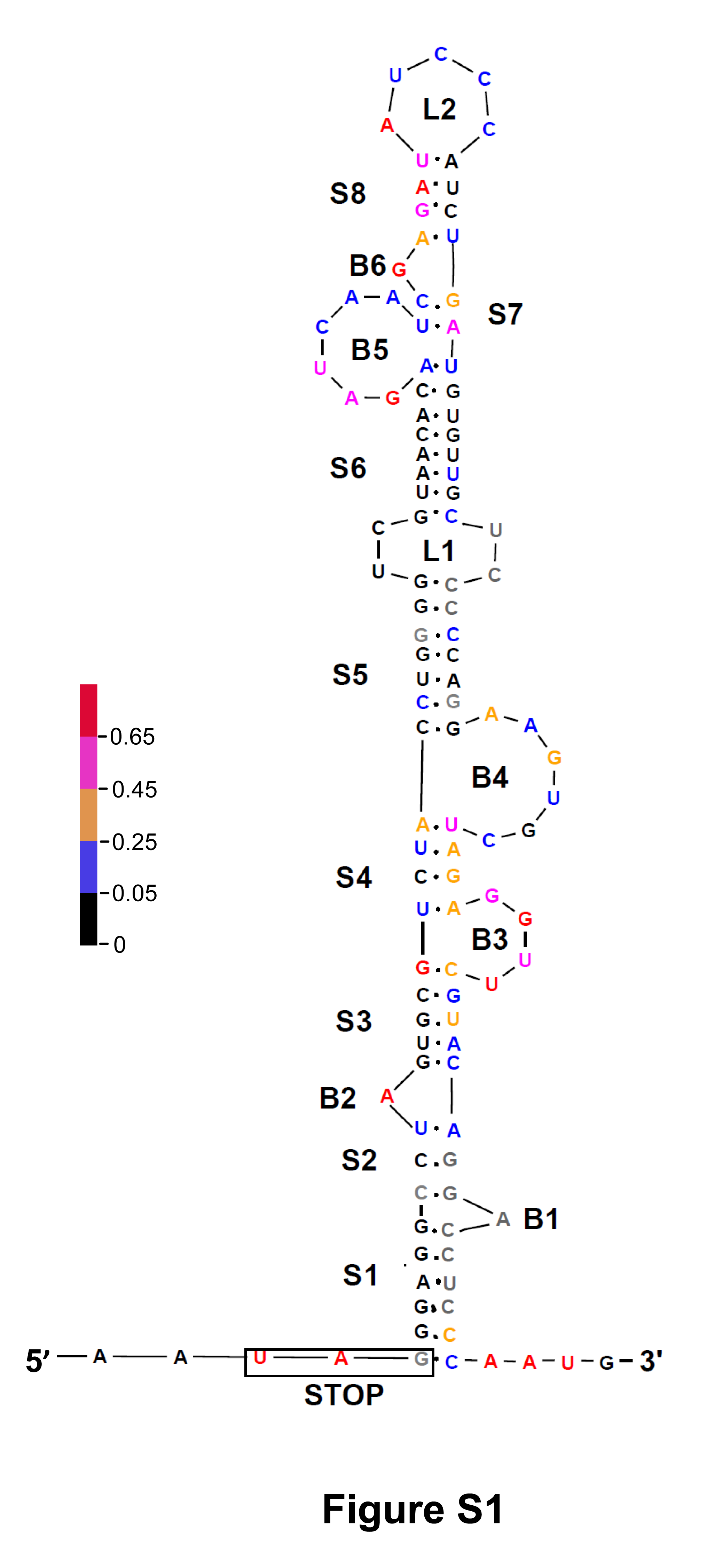

Supplement: Figure S1 — SHAPE analysis of SL-PRTE RNA. SHAPE analysis was performed on the wt CIRV genome and the results were mapped onto the mfold-predicted RNA structure for SL-PRTE. Relative reactivity of each residue is indicated by the color-coded key, with higher values corresponding to increased flexibility. (TIF) [file ppat.1002423.s001.tif]

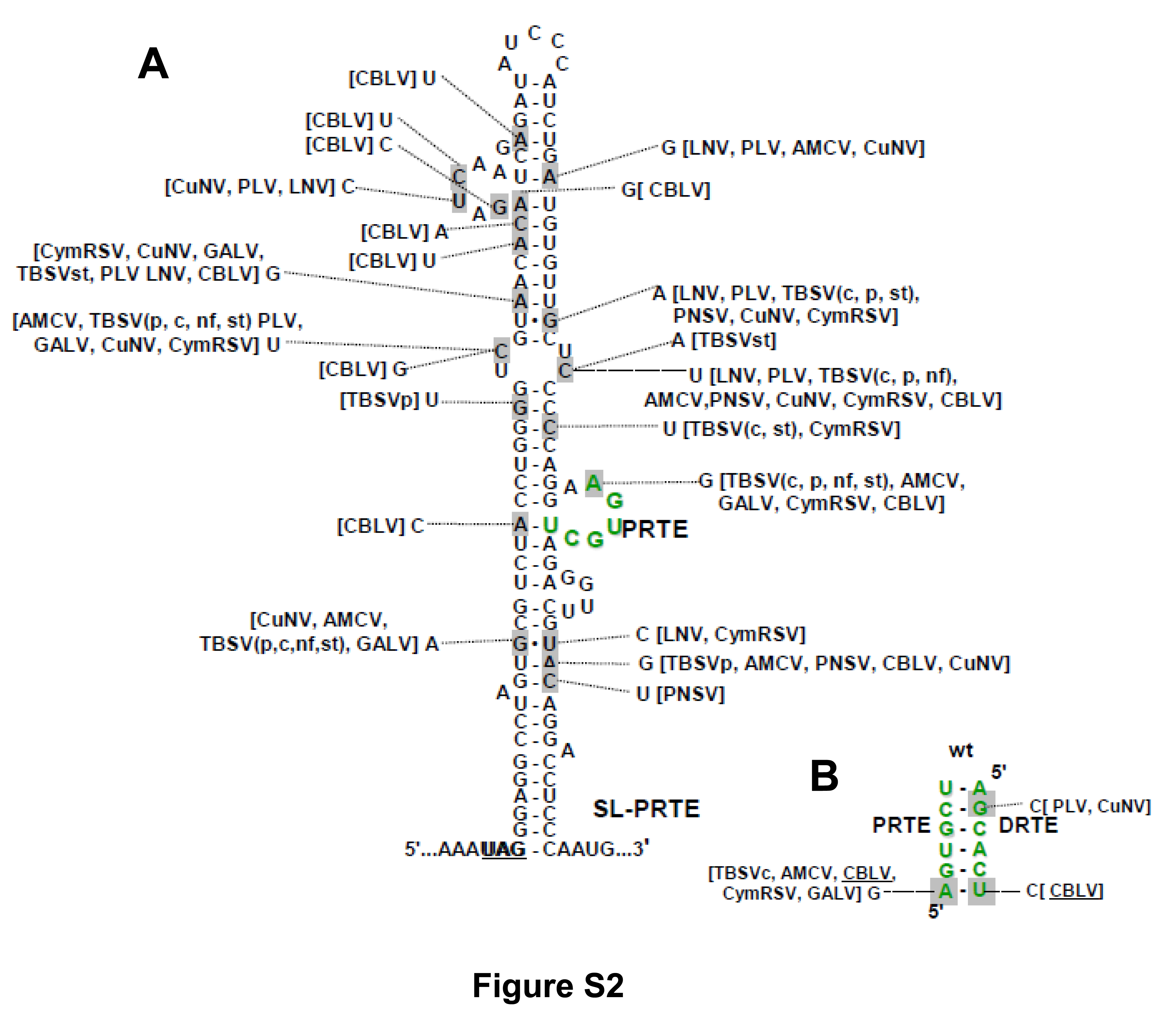

Supplement: Figure S2 — Comparative RNA sequence and secondary structure analysis of SL-PRTE and the PRTE-DRTE interaction. (A) The CIRV SL-PRTE secondary structure and (B) CIRV PRTE-DRTE interaction are shown along with sequence variations found in other tombusviruses. Nucleotides involved in the PRTE-DRTE interaction are shown in green and the p36 stop codon is in bold and underlined. Gray shading indicates CIRV nucleotides that are substituted by different nucleotides in other tombusviruses, with the corresponding identity of the virus indicated in brackets. The virus acronyms are as follows: AMCV, Artichoke mottled crinkle virus: CBLV, Cucumber Bulgarian latent virus; CuNV, Cucumber necrosis virus; CymRSV, Cymbidium ring spot virus; GALV, Grapevine Algerian latent virus; LNV, Lisianthus necrosis virus; PLV, Pear latent virus; PNSV, Pelargonium necrotic spot virus; TBSVc, Tomato bushy stunt virus (cherry isolate); TBSVnf, Tomato bushy stunt virus (nipple fruit isolate); TBSVp, Tomato bushy stunt virus (pepper isolate); TBSVst, Tomato bushy stunt virus (statice isolate). (TIF) [file ppat.1002423.s002.tif]

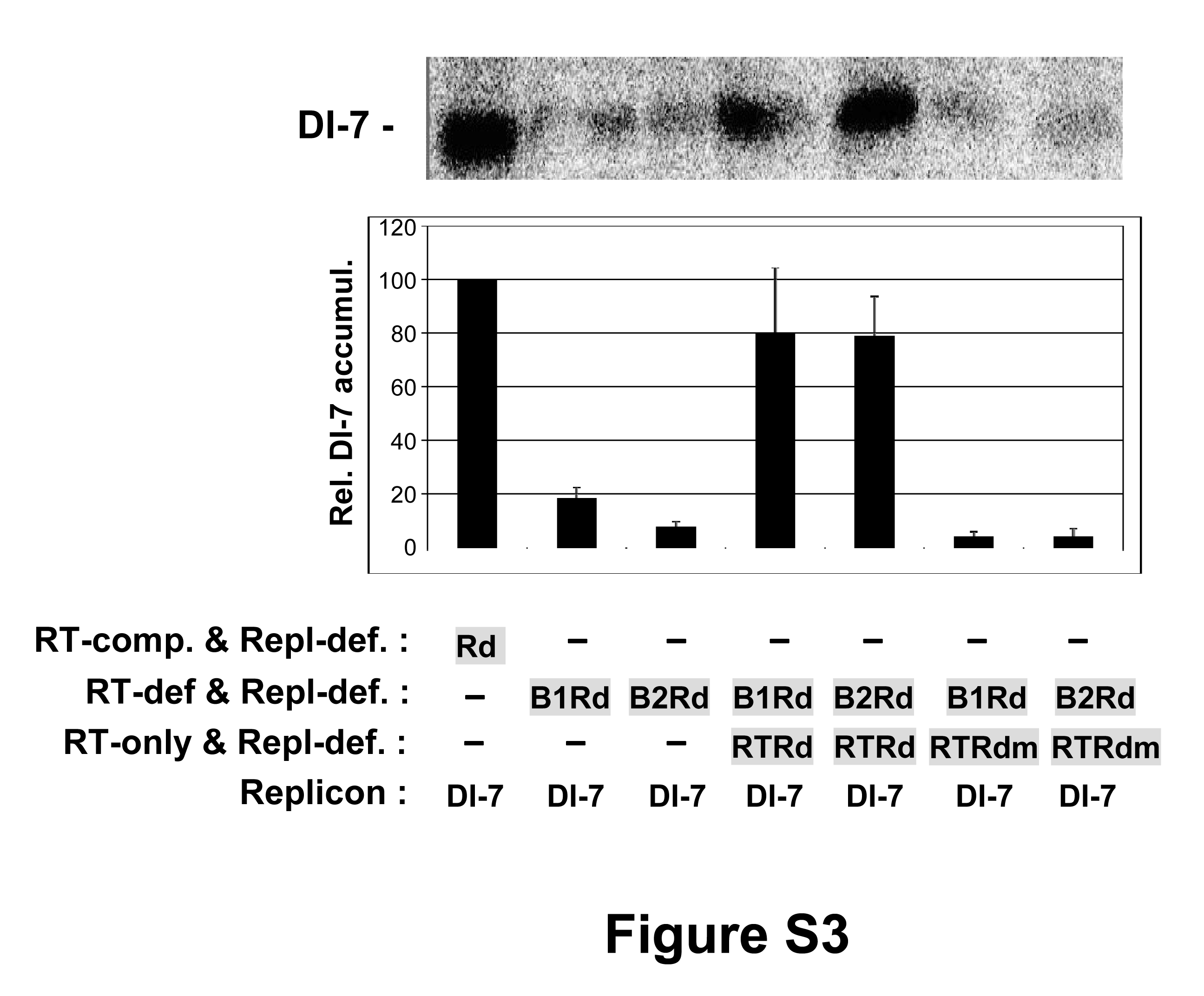

Supplement: Figure S3 — RdRp activity is required to complement RT-deficient mutants. Northern blot analysis and quantification of DI-7 accumulation when cotransfected with non-replicating CIRV genomes in plant protoplasts. The CIRV genomes cotransfected with DI-7 are indicated below each bar in the graph. The mutants tested are described in Figure 4, except for mutant RTRdm, which contains a GDD-to-AAA triple codon substitution in its p95 GDD RdRp motif. DI-7 was analyzed 22 hr post-transfection of plant protoplasts. Northern blot analysis (top) was used to quantify the relative levels of DI-7 accumulation shown in the bar graph (± standard error) that was derived from three independent experiments. (TIF) [file ppat.1002423.s003.tif]

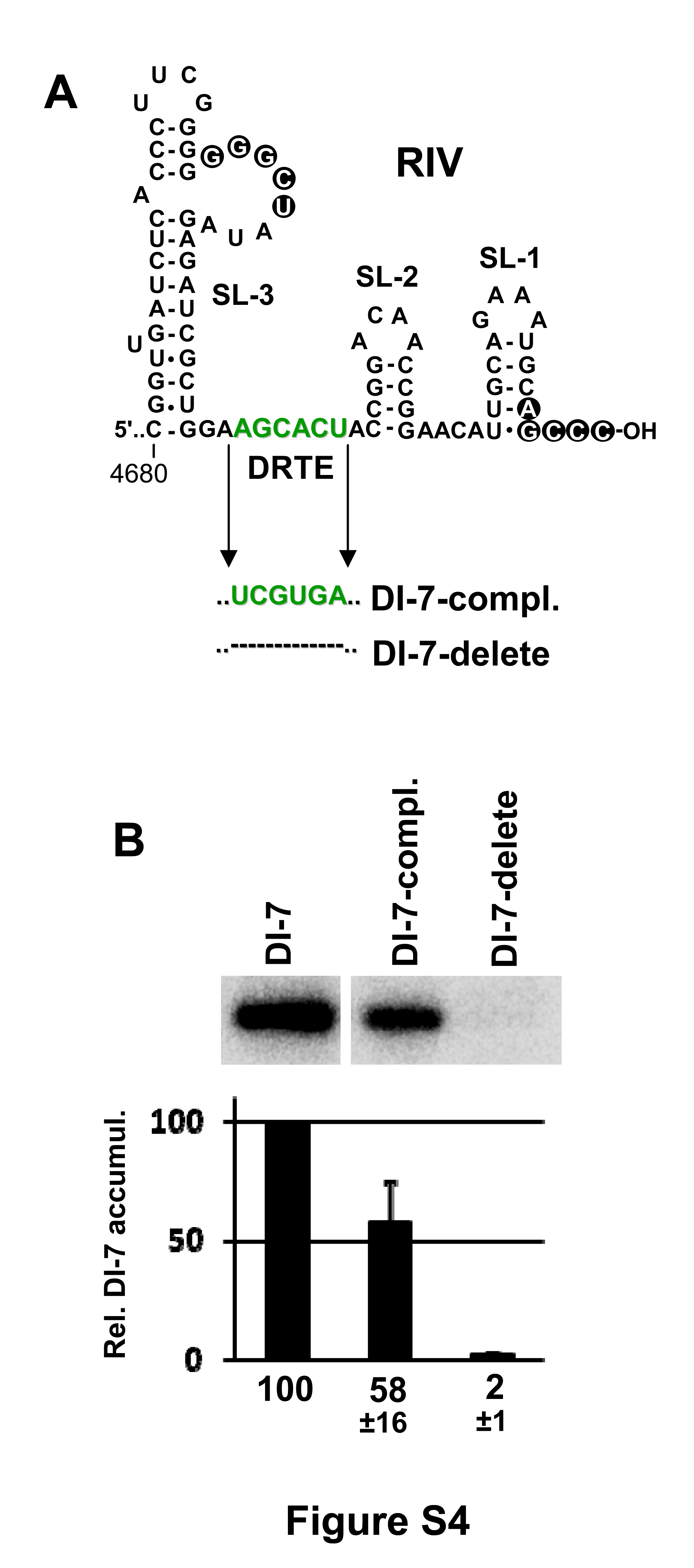

Supplement: Figure S4 — Importance of the DRTE for DI-7 replication. (A) Predicted secondary structure for RIV with the DRTE shown in green. The substitutions and deletion made in the DRTE are shown below the structure. (B) Northern blot analysis and quantification of wt and mutant DI-7 RNA accumulation when cotransfected with wt CIRV in plant protoplasts. DI-7 RNA levels were analyzed and measured by Northern blot analysis 22 hr post-transfection of plant protoplasts. The relative values shown in the bar graph below the lanes correspond to means (± standard error) from three independent experiments. (TIF) [file ppat.1002423.s004.tif]

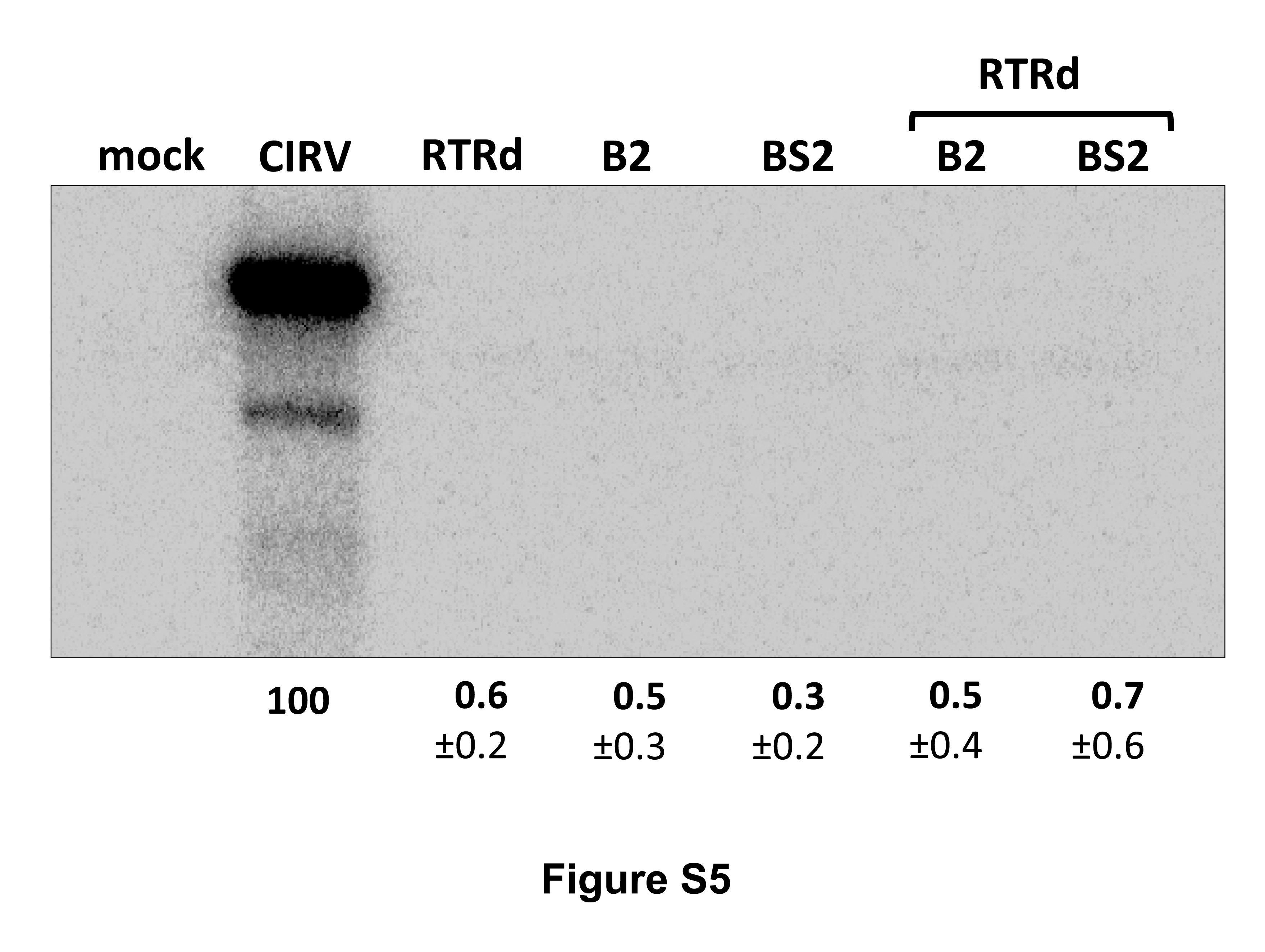

Supplement: Figure S5 — RT-defective genomes are not rescued by p95 supplied in trans. Northern blot analysis and quantification of CIRV genomes analyzed 22 hr post-transfection of plant protoplasts. The identities of the viral genomes are shown above the lanes. Cotransfection of CIRV genome mutants B2 or BS2 with non-replicating RTRd (providing p95) did not lead to rescue of genome replication. The relative values for genome accumulation shown below the lanes correspond to means (± standard error) from three independent experiments. (TIF) [file ppat.1002423.s005.tif]

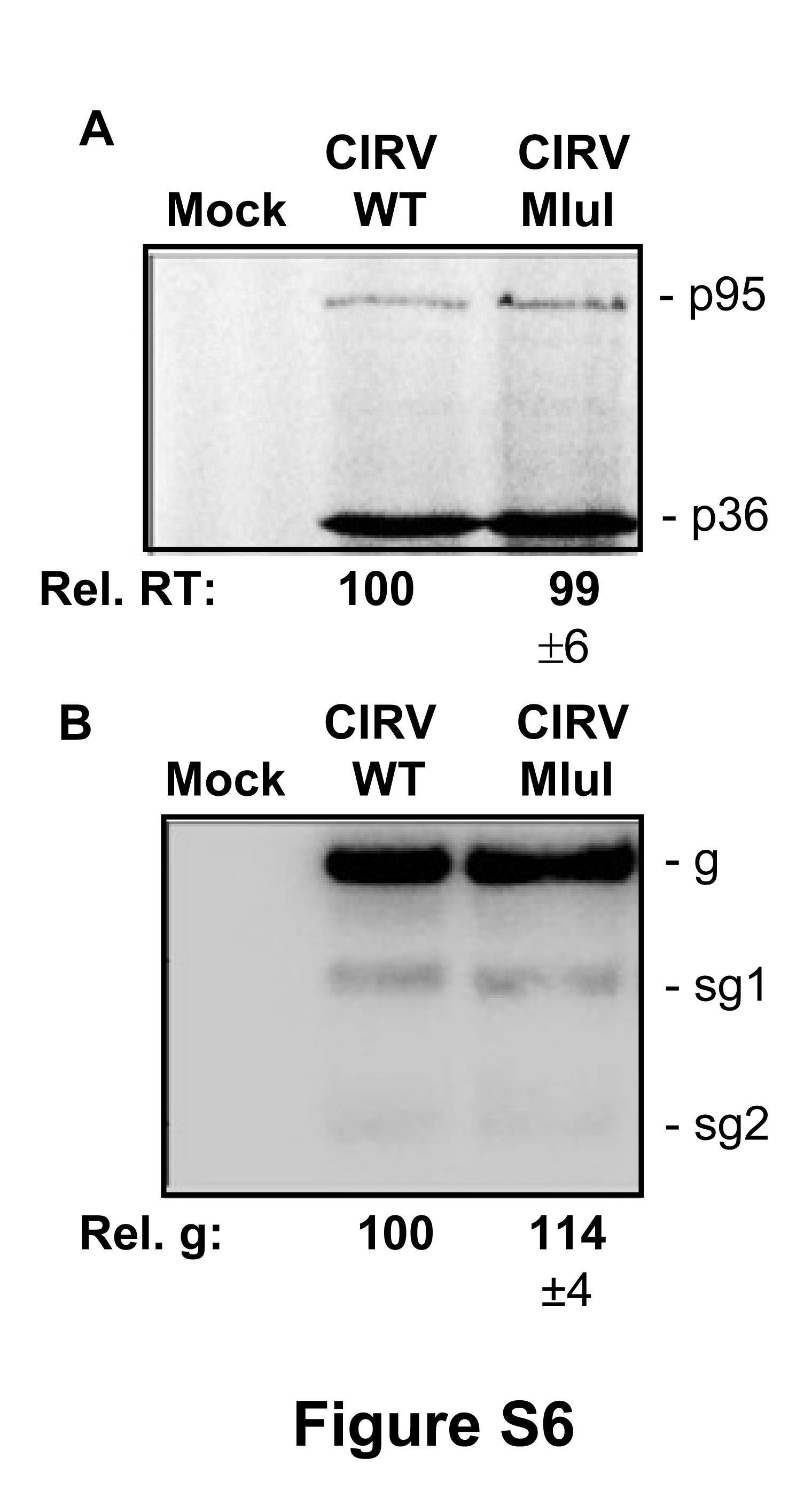

Supplement: Figure S6 — Analysis of CIRV genome containing an engineered MluI restriction. (A) SDS-10%PAGE analysis of p36 stop codon RT from wt CIRV and CIRV containing an MluI restriction site (CIRV MluI). The p95:p36 ratio was determined for each lane, and the relative RT percentages below each lane correspond to means (± standard error) from three independent experiments. (B) Northern blot analysis and quantification of plus-strand accumulation of viral RNAs from CIRV WT and CIRV MluI transfections of plant protoplasts. The positions of the genomic (g) and subgenomic mRNAs (sg1 and sg2) are indicated to the right of the blot. Viral RNAs were analyzed 22 hr post-transfection and the relative values for genomic accumulation below the lanes correspond to means (± standard error) from three independent experiments. (TIF) [file ppat.1002423.s006.tif]
